# Whole genome sequence analyses of eGFR in 23,732 people representing multiple ancestries in the NHLBI trans-omics for precision medicine (TOPMed) consortium

**DOI:** 10.1016/j.ebiom.2020.103157

**Published:** 2021-01-06

**Authors:** Bridget M Lin, Kelsey E Grinde, Jennifer A Brody, Charles E Breeze, Laura M Raffield, Josyf C Mychaleckyj, Timothy A Thornton, James A Perry, Leslie J Baier, Lisa de las Fuentes, Xiuqing Guo, Benjamin D Heavner, Robert L Hanson, Yi-Jen Hung, Huijun Qian, Chao A Hsiung, Shih-Jen Hwang, Margaret R Irvin, Deepti Jain, Tanika N Kelly, Sayuko Kobes, Leslie Lange, James P Lash, Yun Li, Xiaoming Liu, Xuenan Mi, Solomon K Musani, George J Papanicolaou, Afshin Parsa, Alex P Reiner, Shabnam Salimi, Wayne H-H Sheu, Alan R Shuldiner, Kent D Taylor, Albert V Smith, Jennifer A Smith, Adrienne Tin, Dhananjay Vaidya, Robert B Wallace, Kenichi Yamamoto, Saori Sakaue, Koichi Matsuda, Yoichiro Kamatani, Yukihide Momozawa, Lisa R Yanek, Betsi A Young, Wei Zhao, Yukinori Okada, Gonzalo Abecasis, Bruce M Psaty, Donna K Arnett, Eric Boerwinkle, Jianwen Cai, Ida Yii-Der Chen, Adolfo Correa, L Adrienne Cupples, Jiang He, Sharon LR Kardia, Charles Kooperberg, Rasika A Mathias, Braxton D Mitchell, Deborah A Nickerson, Steve T Turner, Vasan S Ramachandran, Jerome I Rotter, Daniel Levy, Holly J Kramer, Anna Köttgen, NHLBI Trans-Omics for Precision Medicine (TOPMed) Consortium, TOPMed Kidney Working Group, Stephen S Rich, Dan-Yu Lin, Sharon R Browning, Nora Franceschini

**Affiliations:** a Department of Biostatistics, University of North Carolina, Chapel Hill, NC, United States; b Department of Mathematics, Statistics, and Computer Science, Macalester College, St. Paul, MN, United States; c Cardiovascular Health Research Unit, Department of Medicine, University of Washington, Seattle, WA, United States; d Division of Cancer Epidemiology and Genetics, National Cancer Institute, National Institutes of Health, Department Health and Human Services, Bethesda, MD, United States; e UCL Cancer Institute, University College London, London WC1E 6BT, United Kingdom; f Altius Institute for Biomedical Sciences, Seattle, WA 98121, United States; g Department of Genetics, University of North Carolina, Chapel Hill, NC, United States; h Center for Public Health Genomics, University of Virginia, Charlottesville, VA, United States; i Department of Biostatistics, University of Washington, Seattle, WA, United States; j Division of Endocrinology, Diabetes and Nutrition, and Program for Personalized and Genomic Medicine, University of Maryland School of Medicine, Baltimore, MD, United States; k Phoenix Epidemiology and Clinical Research Branch, National Institute of Diabetes and Digestive and Kidney Diseases, National Institutes of Health, Phoenix, AZ, United States; l Cardiovascular Division, Washington University School of Medicine, St. Louis, MO, United States; m The Institute for Translational Genomics and Population Sciences, Department of Pediatrics, The Lundquist Institute for Biomedical Innovation at Harbor-UCLA Medical Center, Torrance, CA United States; n Endocrinology and Metabolism, Tri-Service General Hospital Songshan branch, Taipei, Taiwan; o Department of Statistics and Operations Research, University of North Carolina, Chapel Hill, NC, United States; p Endocrinology and Metabolism, National Taiwan University Hospital, Taipei, Taiwan; q National Heart, Lung, and Blood Institute Framingham Heart Study, Framingham, MA, United States; r National Heart, Lung and Blood Institute, Population Sciences Branch, Division of Intramural Research, Bethesda, MD, United States; s Department of Epidemiology, University of Alabama at Birmingham, Birmingham, AL, United States; t Department of Epidemiology, Tulane University School of Public Health and Tropical Medicine, New Orleans, LA, United States; u Division of Biomedical Informatics and Personalized Medicine, Department of Medicine, University of Colorado Anschutz Medical Campus, Denver, CO, United States; v Department of Medicine, University of Illinois, Chicago, IL, United States; w USF Genomics & College of Public Health, University of South Florida, Tampa, FL, United States; x Department of Medicine, University of Mississippi Medical Center, Jackson, MS, United States; y Epidemiology Branch, National Heart, Lung, and Blood Institute, Bethesda, MA, United States; z Division of Kidney, Urologic and Hematologic Diseases, National Institute of Diabetes and Digestive and Kidney Diseases, National Institutes of Health, Bethesda, MA, United States; aa Division of Public Health Sciences, Fred Hutchinson Cancer Research Center, Seattle, WA, United States; bb Epidemiology and Public Health, University of Maryland School of Medicine, Baltimore, MD, United States; cc Endocrinology & Metabolism, Taichung Veterans General Hospital, Taichung, Taiwan; dd Department of Biostatistics, University of Michigan, Ann Arbor, MI, United States; ee Department of Epidemiology, University of Michigan, Ann Arbor, MI, United States; ff Department of Epidemiology, Johns Hopkins Bloomberg School of Public Health, Baltimore, MD, United States; gg Department of Medicine, Johns Hopkins University School of Medicine, Baltimore, MD, United States; hh University of Iowa College of Public Health, Iowa City, IA, United States; ii Department of Statistical Genetics, Osaka University Graduate School of Medicine, Suita 565-0871, Japan; jj Department of Pediatrics, Osaka University Graduate School of Medicine, Suita 565-0871, Japan; kk Department of Allergy and Rheumatology, Graduate School of Medicine, the University of Tokyo, Tokyo 113-8655, Japan; ll Department of Computational Biology and Medical Sciences, Graduate school of Frontier Sciences, The University of Tokyo, Tokyo 108-8639, Japan; mm Laboratory for Statistical Analysis, RIKEN Center for Integrative Medical Sciences, Yokohama 230-0045, Japan; nn Laboratory of Complex Trait Genomics, Department of Computational Biology and Medical Sciences, Graduate School of Frontier Sciences, the University of Tokyo, Tokyo 108-8639, Japan; oo Kidney Research Institute and Division of Nephrology, University of Washington, Seattle, WA, United States; pp Department of Biostatistics and Center for Statistical Genetics, University of Michigan, An Arbor, MI, United States; qq Integrated Frontier Research for Medical Science Division, Institute for Open and Transdisciplinary Research Initiatives, Osaka University, Suita 565-0871, Japan; rr Laboratory for Genotyping Development, RIKEN Center for Integrative Medical Sciences, Yokohama 230-0045, Japan; ss Laboratory of Statistical Immunology, World Premier International Immunology Frontier Research Center (WPI-IFReC), Osaka University, Suita 565-0871, Japan; tt Regeneron Pharmaceuticals, Tarrytown, NY, United States; uu Departments of Epidemiology and Health Services, University of Washington, Seattle, WA, United States; vv College of Public Health, Dean's Office, University of Kentucky, Lexington, KY, United States; ww Human Genetics Center, Department of Epidemiology, Human Genetics, and Environmental Sciences, School of Public Health, The University of Texas Health Science Center at Houston, Houston, TX, United States; xx Human Genome Sequencing Center, Baylor College of Medicine, Houston, TX, United States; yy Department of Biostatistics, Boston University, Boston, MA, United States; zz Geriatrics Research and Education Clinical Center, Baltimore Veterans Administration Medical Center, Baltimore, MD, United States; aaa Department of Genome Sciences, University of Washington, Seattle, WA, United States; bbb Department of Internal Medicine, Mayo Clinic, Rochester, MN, United States; ccc Division of Preventive Medicine and Epidemiology and Cardiology, Boston University School of Medicine, Boston, MA, United States; ddd Department of Public Health Sciences and Medicine, Loyola University Chicago, Maywood, IL, United States; eee Division of Nephrology and Hypertension, Loyola University Chicago, Maywood, IL, United States; fff Institute of Genetic Epidemiology, Faculty of Medicine and Medical Center, University of Freiburg, Freiburg, Germany; ggg Department of Epidemiology, Gillings School of Global Public Health, University of North Carolina, Chapel Hill, NC, United States; § Members listed at the end of the article.

**Keywords:** Whole genome sequencing, Kidney traits, Rare variants, Ancestry-specific variants

## Abstract

**Background:**

Genetic factors that influence kidney traits have been understudied for low frequency and ancestry-specific variants.

**Methods:**

We combined whole genome sequencing (WGS) data from 23,732 participants from 10 NHLBI Trans-Omics for Precision Medicine (TOPMed) Program multi-ethnic studies to identify novel loci for estimated glomerular filtration rate (eGFR). Participants included European, African, East Asian, and Hispanic ancestries. We applied linear mixed models using a genetic relationship matrix estimated from the WGS data and adjusted for age, sex, study, and ethnicity.

**Findings:**

When testing single variants, we identified three novel loci driven by low frequency variants more commonly observed in non-European ancestry (*PRKAA2*, rs180996919, minor allele frequency [MAF] 0.04%, *P* = 6.1 × 10^−11^; *METTL8*, rs116951054, MAF 0.09%, *P* = 4.5 × 10^−9^; and *MATK*, rs539182790, MAF 0.05%, *P* = 3.4 × 10^−9^). We also replicated two known loci for common variants (rs2461702, MAF=0.49, *P* = 1.2 × 10^−9^, nearest gene *GATM*, and rs71147340, MAF=0.34, *P* = 3.3 × 10^−9^, *CDK12*). Testing aggregated variants within a gene identified the *MAF* gene. A statistical approach based on local ancestry helped to identify replication samples for ancestry-specific variants.

**Interpretation:**

This study highlights challenges in studying variants influencing kidney traits that are low frequency in populations and more common in non-European ancestry.

**RESEARCH IN CONTEXT**

**Evidence before this study**

Several loci have been identified for estimated glomerular filtration rate (eGFR) in genome-wide association studies. Genetic factors that influence kidney traits have been understudied for low frequency and ancestry-specific variants.

**Added value of this study**

The main findings of this study are the identification of ancestry-specific rare variants associated with eGFR either individually or in aggregate units within a gene. We also showed the utility of estimating ancestry-specific allele frequencies for rare sequencing variants using local ancestry to identify ancestry-related replication samples when using multi-ethnic studies. This study also highlights challenges for the study of rare/low frequency variants in multi-ethnic studies, including finding suitable samples for replication of ancestry-specific variants.

**Implications of all the available evidence**

Rare and low frequency variants are more likely to be population-specific and their genetic contribution to eGFR variation is mostly unknown. Our study provides important information for future WGS studies of rare SNVs for kidney traits, with implications for study design of variant discovery and replication, particularly when studying diverse ancestry populations.

Alt-text: Unlabelled box

## 1 Introduction

Reduced kidney function, assessed with the estimate glomerular filtration rate (eGFR), defines chronic kidney disease (CKD). Low eGFR is associated with cardiovascular disease morbidity [1], mortality [2,3], poor quality of life and high health care costs for its treatment [4]. CKD has a high burden among non-European ethnic groups [5]. In the U.S., the burden of CKD in African American is attributed in part to the presence of ancestry-specific genetic variants, i.e., *APOL1* high-risk genotypes [6]. Genetic factors and underlying pathways influencing eGFR in populations can provide insights into CKD occurrence, mechanistic pathways, and downstream complications.

Genetic studies that included ethnically diverse populations have yielded important gains in gene discovery and have advanced fine mapping by leveraging differences in allele frequencies and in coinheritance of genetic variants across ancestral groups [7,8]. In addition, substantial evidence indicates that ancestry-specific genetic variants contribute to CKD [6,9]. The current study expands on prior genetic studies of kidney loci through interrogation of rare and low frequency variants from whole genome sequencing (WGS) in the National Heart Lung and Blood Institute's Trans-Omics for Precision Medicine (TOPMed) program. We aimed to understand a role of rare and low frequency variants that individually or in aggregate influence eGFR, and to identify ancestry-specific genomic regions associated with eGFR in African Americans and Hispanics/Latinos through admixture mapping. Using a newly described statistical approach based on local ancestry, we estimated ancestry-specific allele frequencies for rare sequencing variants and showed their utility for identifying ancestry-related replication samples.

## 2 Methods

### 2.1 Ethics statement

All human research was approved by the relevant institutional review boards and conducted according to the Declaration of Helsinki. All participants provided written informed consent.

### 2.2 Study design and participants

The study included 23,732 participants from ten studies with phenotype data and WGS from the TOPMed Freeze 5b, for five racial/ethnic groups: European Americans, African Americans, East Asians, Hispanic/Latinos, and Native Americans. We followed TOPMed guidelines when reporting race/ethnicity and ancestry (WEB Resources). Admixture mapping analyses included a subset of 9,479 admixed African Americans and Hispanics/Latinos. The following studies contributed data: Old Order Amish, Atherosclerosis Risk in Communities (ARIC), Framingham Heart Study (FHS), Genetic Epidemiology Network of Arteriopathy (GENOA), Genetic Epidemiology Network of Salt Sensitivity (GenSalt), Genetic Study of Atherosclerosis Risk (GeneSTAR), Hypertension Genetic Epidemiology Network (HyperGEN), Jackson Heart Study (JHS), Multi-Ethnic Study of Atherosclerosis (MESA) and Women's Health Initiative (WHI). Demographic, clinical data and kidney phenotypes were obtained from study clinical visits.

### 2.3 Phenotyping procedures

We performed centralised harmonization of the phenotype and eGFR was calculate using the serum creatinine-based Chronic Kidney Disease Epidemiology Collaboration (CKD-EPI) equation [10]. For studies with serum creatinine assayed before 2009 using a Jaffe assay, we multiplied the serum creatinine value to 0.95. The CKD-EPI eGFR estimation uses a race term for black race to account for biological variations in non-GFR determinants. This equation has been widely used in both research and clinical care, although some concerns have been raised related to race component of the equation [11]. To account for differences in trait distribution by study and among ethnic groups, eGFR was inverse normalised within study and racial/ethnic groups and then rescaled to recover original trait variance [12]. Therefore, results are reported using units of ml/min/1.73 m^2^.

### 2.4 Whole genome sequencing data generation and quality control

Contributing studies had WGS from TOPMed Freeze 5b. WGS was performed at an average depth of 38x using DNA from blood as previously reported^4^. Processing of whole genome sequences was harmonised across genomic centres using a standard pipeline (see URL in the Web Resources section). Briefly, participants were sequenced at the Broad Institute, the Northwest Genomic Center at the University of Washington, and the New York Genome Center. GeneSTAR samples were sequenced at Macrogen and Illumina. Central quality control and variant calling was performed jointly at the University of Michigan Informatics Resource Center. Further quality control that focused on sample identity was performed at the University of Washington Data Coordinating Center. All methods are described on the dbGaP website at: https://goo.gl/ntuJbR. After site level filtering, TOPMed freeze 5b consisted of ~438 million single nucleotide variants (SNVs) and ~33 million short insertion-deletion (indel) variants. Most indels were singleton or rare, with only 1–2% with allele frequency > 1%. Read mapping was done using the 1000 Genomes Project reference sequence versions for human genome build GRCh38. Functional annotations were performed using the WGSA Annotator [13], and WGSAParsr was used to generate simplified WGSA annotation files and variant grouping files for gene-based aggregate tests (see URL in the Web Resources section). Principal components (PC) of ancestry were estimated among all samples using PC-Relate and PC-AiR [14, 15]. We used the Omics Analysis, Search and Information System (OASIS) based on TOPMed data for the included individuals to calculate linkage disequilibrium among SNVs and to plot genomic regions (see URL in the Web Resources section).

### 2.5 Statistical methods

#### 2.5.1 Single variants association analysis and gene-based collapsing analysis

Association analyses were performed in a cloud computing environment under DNAnexus [16] (see URL in the Web Resources section).

*Single variant association test.* We fitted a linear mixed model using covariates of age, sex, and categories of study, race/ethnicity, and case-control status as needed. To account for genetic similarity among subjects, we used the genetic relationship matrix estimated from the WGS data from PC-Relate to specify the random effects covariance structure. We allowed for heterogeneous residual variance components, and grouped subjects by study, race/ethnicity, and case-control status. We used the Wald test for single variant association analyses of 43,622,178 autosomal variants filtered for a minor allele count > 10. The significance threshold was *p* < 5.0 × 10^−9^, which has been determined to be the appropriate genome-wide threshold for sequencing studies [17, 18]. We estimated the phenotypic variance explained (PVE) by each variant and their joint PVE using methods described in Supplemental material. Although this study is focused on rare and low frequency variants, we also examined the association of previously reported common variants at eGFR loci (Genome Catalogue, see URL in the Web Resources section) and the presence of secondary associations at the loci that were genome-wide significant in our single variant analyses using conditional analyses. The conditional analyses used the most significant SNV in our data as a covariate and examined if there were additional SNVs with a p-value lower than the index SNV within a window of 1 Mbase of the index SNV.

*Gene-based association tests.* For gene-based tests, variants were aggregated by GENCODE genes (v24). Variants within a gene were filtered to retain a set of rare variants (minor allele frequency [MAF] < 1%) that were predicted as loss-of-function variants (LoF), protein altering small deletions/insertions (indels) or synonymous SNVs which have a deleterious functional annotation (FATMM-MKL score>0.5 or MetaSVM score > 0 for missense SNVs). Variants in a 5 kb window promoter region (upstream of transcription start site [19] and in a FANTOM5 [Functional ANalyses Through Hidden Markov models] peak) [18] and variants at the first intron of genes were also included. Genes with at least 10 individuals with at least one copy of any alternative allele were included. We performed both burden and SKAT tests and used a conservative significance threshold of *p* < 1.6 × 10^−6^ based on Bonferroni correction for two tests on each of 16,054 genes included in analyses. To identify the contribution of one or more variants within genes with a gene-based significant association, we tested the association of each single variant within the aggregate gene unit. We performed leave-one-variant-out analyses with variants aggregated within a gene for gene-based tests.

#### 2.5.2 Admixture mapping

These analyses included only self-identified African American, African Caribbean, or Hispanic/Latino TOPMed participants (*n* = 20,048) of which 9,479 had eGFR data. The reference panel for local ancestry inference included 37 African, 35 European, and 20 Native American individuals with phased sequence data available from the Simons Genome Diversity Project (SGDP) [20]. After removing very low frequency variants (minor allele count < 2 in SGDP or < 5 in TOPMed), 9,137,968 autosomal SNVs remained for analysis. We used the HapMap genetic map [21], lifted over to build 38, to estimate genetic positions for each variant, which was needed for inferring local ancestry and to estimate the significance threshold using Significance Threshold Estimation for Admixture Mapping (STEAM). The various maps are highly correlated at the scale that is relevant for admixture mapping (Mbase) [22] and our prior studies have shown no differences when comparing two different choices of genetic maps for inferring local ancestry [23]. We inferred the number of alleles inherited from African, European, and Native American ancestral populations for each admixed individual using RFMix (version 1.5.4) with a window size of 0.1 cM. Generations since admixture (6 for African American samples and 10 for Hispanic/Latino samples) were chosen to reflect estimates from previous studies [24, 25]. To estimate admixture proportions for each individual, we calculated the genome-wide average local ancestry. We used an iterative procedure to estimate kinship coefficients adjusted for population structure and admixture, which were used in our linear mixed model to adjust for relatedness in admixture mapping. In the final step of this iterative procedure, we used our estimated admixture proportions in place of principal components.

We performed admixture mapping using a linear mixed model (GENESIS) on each ancestral group (African, European, and Native American) separately [26]. eGFR was the outcome variable. Models were adjusted for sex, age, study and race/ethnic group (African American or Hispanic/Latino) and admixture proportions as fixed effects, and ancestry-adjusted kinship as a random effect. We allowed for heterogeneous variance within groups defined by study and race. To account for multiple testing, we used the genome-wide threshold of *p* < 5.4 × 10^−6^, estimated using STEAM [23]. As secondary analyses, admixture mapping was conducted separately in the African American and Hispanic/Latino subjects; significance thresholds were *p* < 1.6 × 10^−5^ (testing just the African ancestral component) and *p* < 3.5 × 10^−6^ in the African American and Hispanic/Latino subsets, respectively.

#### 2.5.3 Estimating ancestry-specific allele frequencies

We used our local ancestry calls to estimate ancestry-specific allele frequencies for loci of interest using the recently developed method Ancestry-Specific Allele Frequencies Estimation (ASAFE). RFMix only infers local ancestry at loci that are present in both the admixed sequence data and the reference panel, so inferred local ancestry will not be available at any loci that are not present in SGDP. However, because local ancestry segments extended over multiple loci, we can fill in the missing local ancestry calls at a locus of interest with reasonable confidence by looking at the inferred local ancestry at neighbouring loci. We can then use the local ancestry calls to estimate the ancestry-specific allele frequency for each ancestral population at a locus by calculating the frequency of the allele across haplotypes in our sample with local ancestry assigned to each ancestral population (African, European, and Native American). To account for uncertainty in the phase of genotypes relative to the local ancestry calls (particularly at loci where local ancestry was not inferred directly by RFMix), we used the EM algorithm approach implemented in the ASAFE program [27]. We ran ASAFE using local ancestry calls for the 9,479 subjects included in our admixture mapping analysis.

#### 2.5.4 Replication

Replication was performed using ancestry-specific allele frequency information. For East Asian SNVs, we used data from the Rare Variants for Hypertension in Taiwan Chinese (THRV) study, which was sequenced in the TOPMed freeze 8 using methods described above. Additional replication for East Asians were obtained from a WGS of 1,524 participants (32%, women, mean age 49.5 years, mean eGFR 102.5 ml/min/1.73 m^2^) from the BioBank Japan Project (BBJ). BBJ is a multi-institutional hospital-based study that collaboratively collected DNA and serum samples from the participants, mainly of Japanese ancestry, with a diagnosis of any of 47 diseases [28, 29]. Participants on dialysis, and those with serum creatinine level outside of three times of interquartile range of upper/lower quartile were excluded. WGS had an average depth of 25.9x as described elsewhere [30]. Rank-based inverse transformation of the eGFR residuals after an adjustment for age, sex, and 47 disease affection status was used as phenotype in single variant and gene-based analyses with an adjustment for 20 top principal components as covariates. For the gene-based analysis for *MAF* gene in the BBJ, 34 variants comprising 4 nonsynonymous SNVs with FATHMM-MKL score>0.5 or MetaSVM score>0, and 30 variants within the first intron were tested. Burden test was conducted using rvtest software and SKAT and SKAT-O using EPACTS software.

For replication of the Amerindian ancestry variant at chromosome 19, we used data from the Hispanic Community Health Study/Study of Latinos (HCHS/SOL), which was genotyped using a custom Illumina array and imputed to the TOPMed Freeze 5b multi-ethnic reference panel. HCHS/SOL analyses were performed among individuals with higher Amerindian ancestry proportion (Mainland sample, *n* = 6,767 individuals) [9]. In addition, we attempted to replicate our findings in two cohorts of southwest American Indians collected by the Intramural National Institute of Diabetes and Digestive and Kidney Diseases (NIDDK) program; namely, participants in a community-based longitudinal study who are predominately of Pima Indian heritage, and participants from the Family Investigation of Nephropathy and Diabetes (FIND), a study of diabetes and diabetic nephropathy in adults. The SNV rs539182790 was direct genotyped using Taqman on Demand in these southwestern American Indian studies and statistical analyses followed the same protocols from TOPMed.

#### 2.5.5 Bioinformatics

We performed a look-up of genomic coordinate overlap (hg38) in the Roadmap Epigenomics and the Encyclopedia of DNA Elements (ENCODE) consortium data [31, 32] across different tissues and samples. All datasets included had been released and passed quality control by respective consortia.

### 2.6 Role of funders

The funders had no role on the study design, execution or interpretation of findings.

## 3 Results

**Fig. 1:**
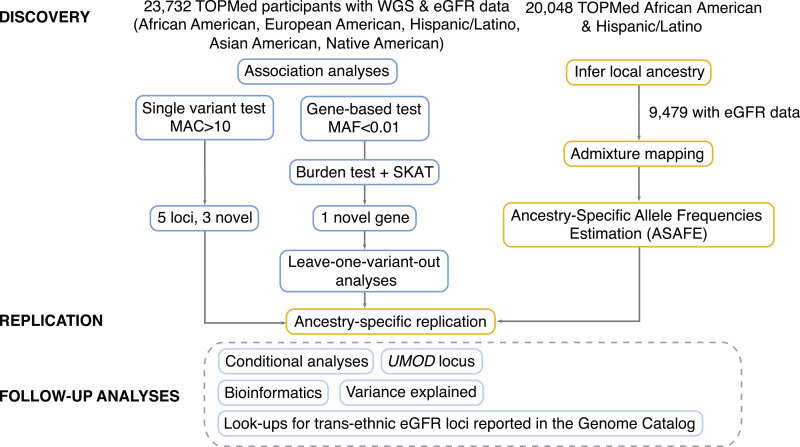
Study design and approaches for discovery, replication and follow-up studies.

Study design is shown in Fig. 1 and the characteristics of participants in **Supp Table 1**. The study comprised up to 23,732 TOPMed participants from 10 multi-ethnic studies and five racial/ethnic groups (36% African Americans, 50% European Americans, 9% East Asians, 5% Hispanics/Latinos and 0.2% American Indians)**.**

### 3.1 Single variant association results

**Fig. 2:**
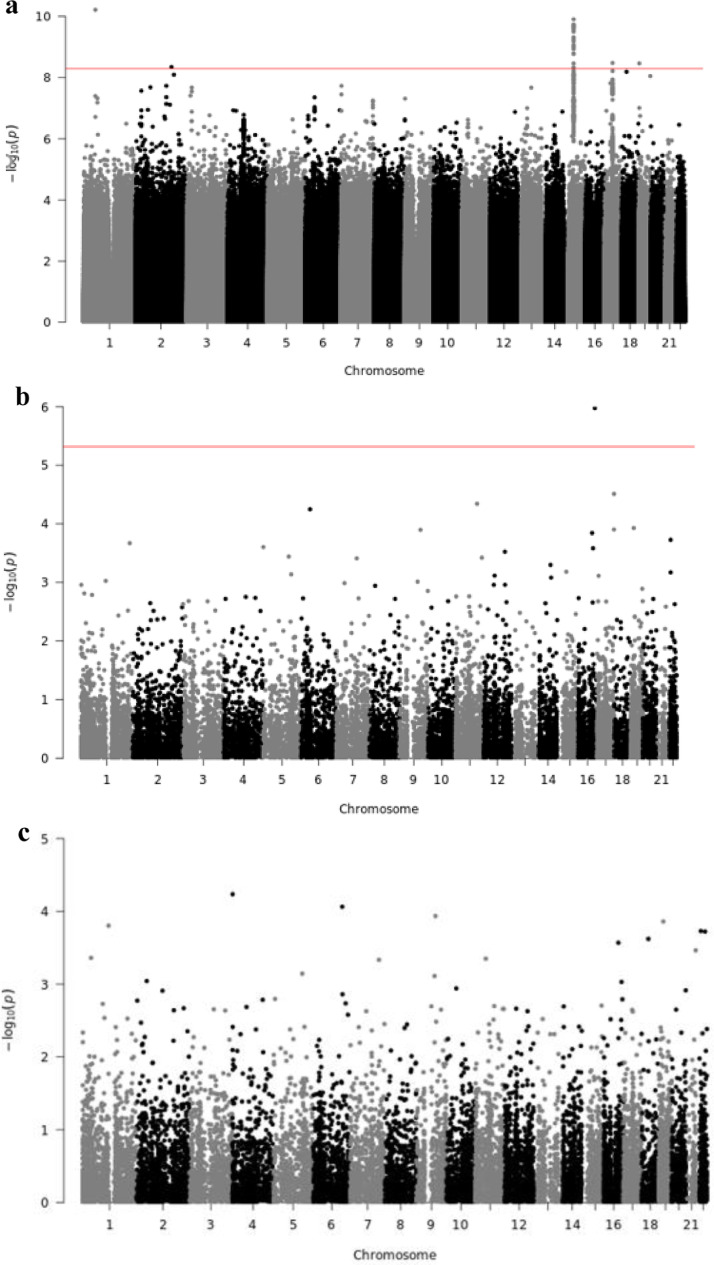
Manhattan plot for (a) WGS single-variant test, (b) SKAT test, and (c) Burden test. The horizontal line represents the significance threshold.

**Fig. 3:**
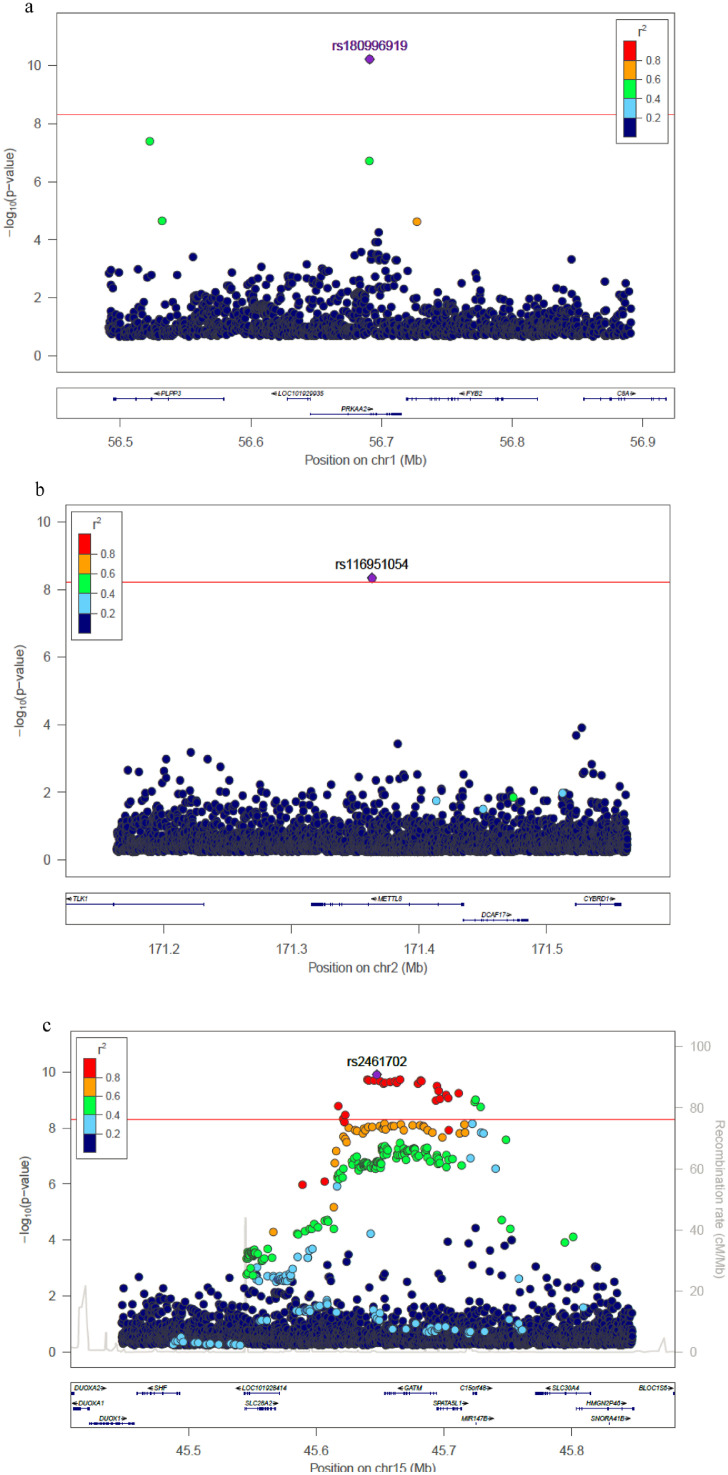

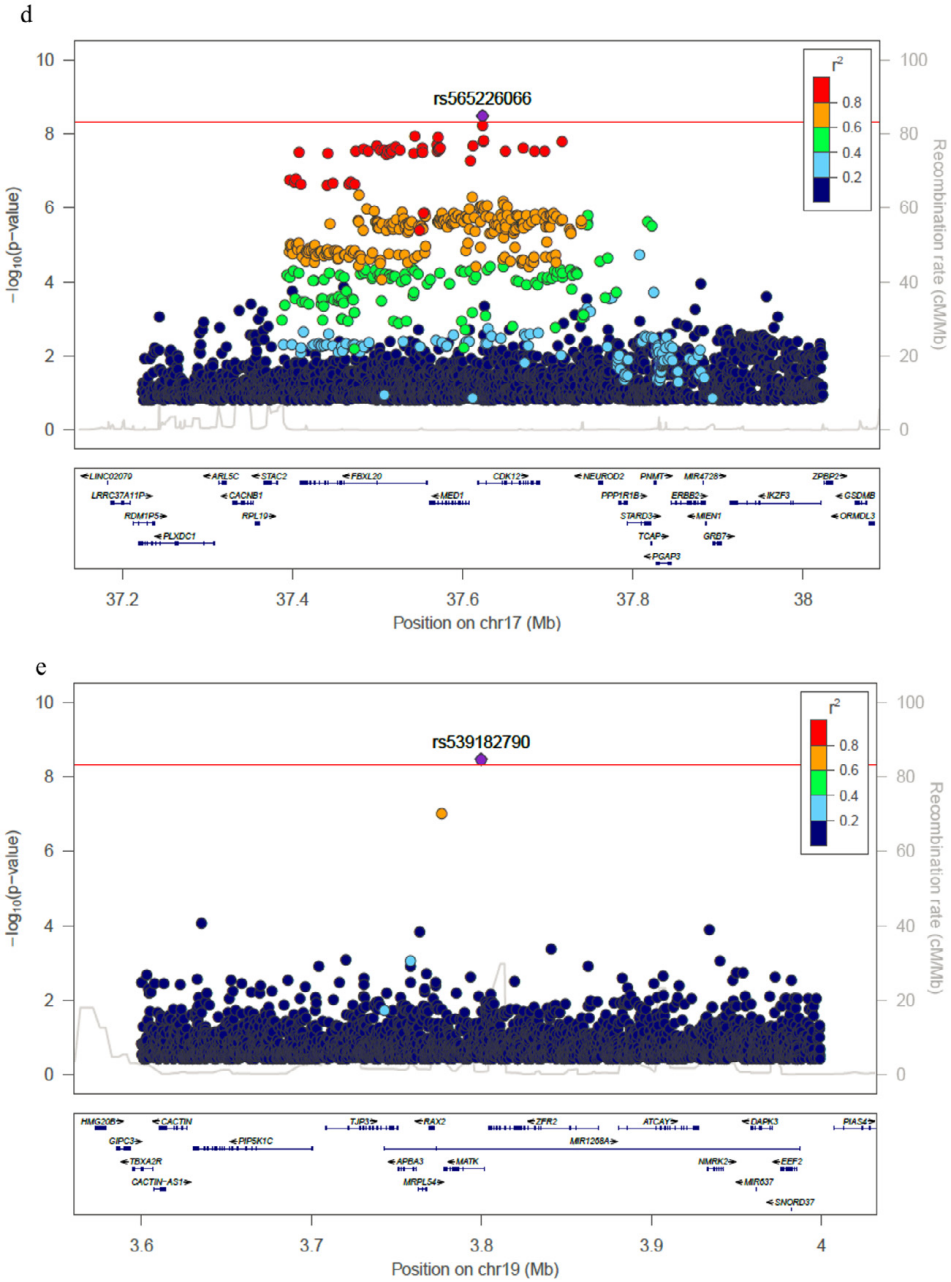
Regional plots for significant associated loci in single variant analyses. (a) Chromosome 1 locus; (b) Chromosome 2 locus; (c) Chromosome 15 locus; (d) Chromosome 17 locus; (e) Chromosome 19 locus. Each point represents a SNV, plotted with their *p*-value (on a -log_10_ scale) as a function of genomic position (NCBI build 38). The index variant is represented by the purple symbol. The color coding of all other SNPs indicates LD with the index variant in haplotypes inferred from the TOPMed data: red *r*^2^≥0.8; gold 0.6 ≤ *r*^2^<0.8; green 0.4 ≤ *r*^2^<0.6; cyan 0.2 ≤ *r*^2^<0.4; blue *r*^2^<0.2; gray *r*^2^ unknown. The horizontal line represents the significance threshold.

Single variant analyses included the 23,732 participants, which were analysed together using a genetic relationship matrix to account for genetic similarities among subjects. Statistical analyses accounted for differences in the eGFR distribution observed in these groups. The quantile-quantile (Q-Q) plot for the WGS analysis is shown in **Supp Fig 1a** (genomic control lambda 1.005). Genome-wide significant associations were identified on chromosomes 1, 2, 15, 17, and 19 (Figure 2 Figure 3) **and**. The variants most significantly associated with eGFR were located on chromosomes 1 (rs180996919, MAF=0.04%, *P* = 6.1 × 10^−11^, PVE 0.2%, intronic to *PRKAA2*), chromosome 2 (rs116951054, MAF=0.09%, *P* = 4.5 × 10^−9^, PVE 0.1%, intronic to *METTL8*) and chromosome 19 (rs539182790, MAF= 0.06%, *P* = 3.4 × 10^−9^, PVE 0.1%, intronic to *MATK*), were all rare and combined explained 0.5% of the trait variance (Table 1). Two variants overlap epigenomic annotations in tissues from the Roadmap Epigenomics Consortium: rs116951054 (DNAse I hypersensitive sites [DHS], H3K4me1 kidney) and rs180996919 (DHS, H3K4me1 muscle). The variants significantly associated with eGFR on chromosomes 15 (rs2461702, MAF=0.49, *P* = 1.2 × 10^−9^, nearest gene *GATM*) and 17 (rs71147340, MAF=0.34, *P* = 3.3 × 10^−9^, intronic indel to *CDK12*) were previously reported [33]. Conditional analyses on identified variants did not reveal additional independent associations at the *GATM* or *CDK12* loci (**Supp Fig**2).

**Table 1:** Significant findings for single trait genome-wide association of eGFR.

Chr:position (hg38)	SNV/indel	Coded allele/Other	Minor allele		Beta (SE) (Coded allele)	*p*	Gene	Function
			Freq Count					
1:56,690,933	rs180996919	C/G	0.0004	19	−14.56 (2.2)	6.1 × 10^−11^	*PRKAA2*	Intronic
2:171,363,037	rs116951054	A/C	0.0009	41	9.10 (1.55)	4.5 × 10^−9^	*METTL8*	Intronic
15:45,355,229*	rs2461702	G/A	0.49	23,170	−0.63 (0.10)	1.2 × 10^−10^	*GATM*	Intergenic
17:39,466,919*	rs71147340	C/CT	0.34	16,247	−0.57 (0.10)	3.3 × 10^−9^	*CDK12*	Intronic
19:3,799,817	rs539182790	G/GGT	0.0005	26	−9.69 (1.64)	3.4 × 10^−9^	*MATK*	Intronic

SNV, single nucleotide variant. Significance threshold 5.0 × 10^−9^; betas are eGFR change in ml/min/1.73 m^2^ for each additional copy of the coded allele. Freq, frequency of coded allele.

⁎ Previously reported loci. Regulatory annotation for rs180996919 (DHS, H3K4me1 muscle) and rs116951054 (DHS, H3K4me1 kidney).

### 3.2 Gene-based test results

**Fig. 4:**
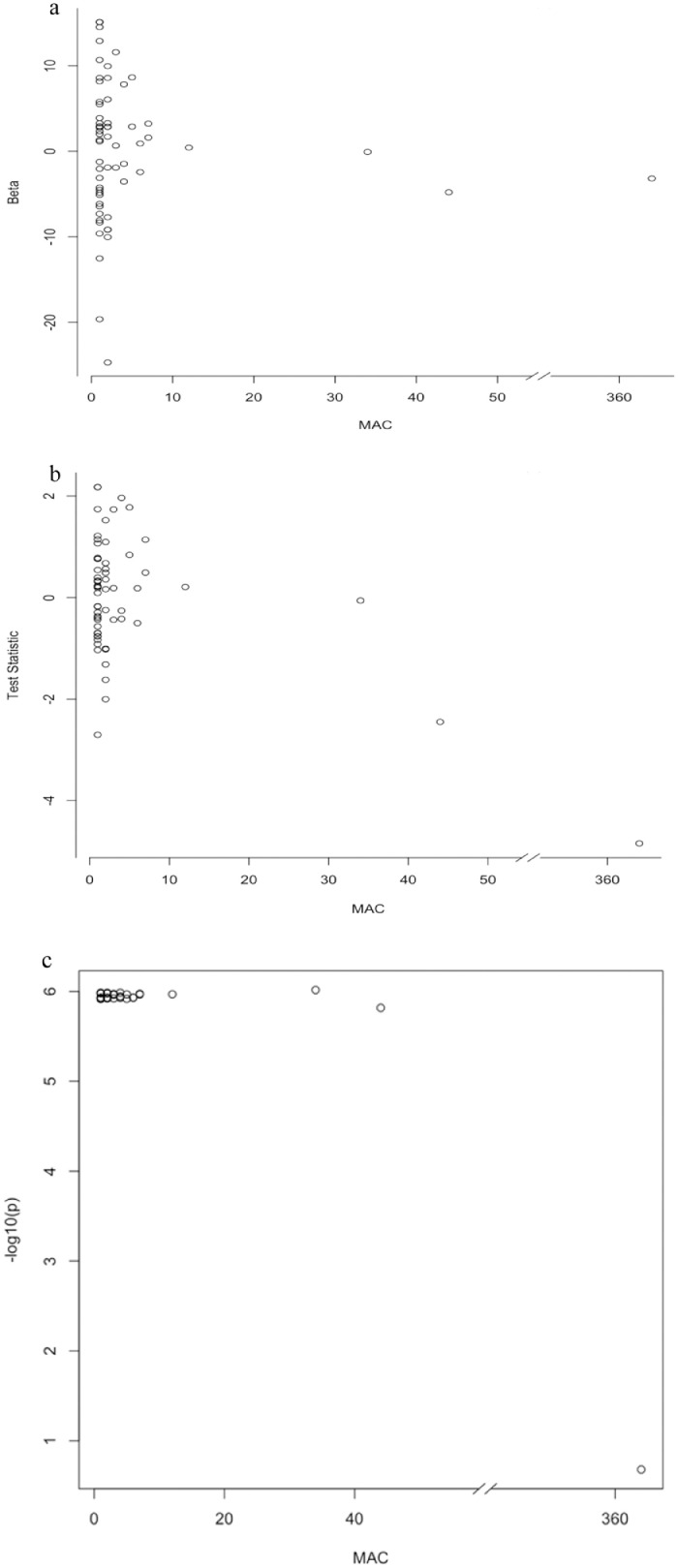
Results from single variant analyses for ENSG00000178573 (*MAF*). (a) Effect estimates by minor allele count (MAC). (b) Test statistic by MAC. (c) Results from the leave-one-variant-out analyses at this gene. Note that rs1230233783 is the influential SNV in the SKAT gene-based analyses.

We next tested variants aggregated within genes (*n* = 16,054 genes) using a burden test that combined all variants in a score test, and the SKAT test, which allows for differences in the direction of effects for rare variants. Q-Q plots are shown in **Supp Fig 1b-c**, respectively. Although the burden tests were not significant, the SKAT analyses identified a significant association for the *MAF* gene, reflecting differences in these approaches for testing rare variants (**Supp Table 2**). Sixty-one variants including 32 singletons contributed to the *MAF* gene-based association in SKAT analysis. To investigate the contribution of single variants to the association at this gene, we performed single variant association analyses for each variant in the gene and identified a missense variant (rs1230233783, p.His191Tyr, AA 191, MAF=0.008) contributing to most of the association with eGFR (*P* = 1.27 × 10^−6^). The SKAT analyses using a leave-one-variant-out strategy supported the strong contribution of rs1230233783 to the gene-based association (Figure 4**, a-c**). This variant also overlaps epigenomic annotations (H3K27ac ENCODE data). Additional genes identified in gene-based analyses are shown in **Supp Table 2**.

### 3.3 Admixture mapping and estimating ancestry-specific allele frequencies

Local ancestry determination and complete phenotype information were available for 9,479 admixed individuals (8,303 African Americans and 1,176 Hispanic/Latinos of 23,732 TOPMed participants with eGFR (**Supp Table 1**). The inferred global ancestry proportions for each African American and Hispanic/Latino individual and the averages (and ranges) of African, Native American and European ancestries are shown in **Supp Fig 3**. These results showed a large variation in ancestry proportions in Hispanics/Latinos in our data. There were no genome-wide significant associations identified using overall sample (**Supp Fig 4**) or separately by African American (**Supp Fig 5**) or Hispanic/Latino ancestry (**Supp Fig 6**).

Local ancestry calls were used to estimate ancestry-specific allele frequencies and rs539182790 allele at *MATK* was exclusively present in the Native American ancestral population (MAF = 0.02) and not in African (MAF = 3.1 × 10^−10^) or European (MAF = 7.9 × 10^−4^) ancestral populations **(**Table 2).

**Table 2:** Estimated ancestry-specific allele frequencies for SNVs identified in single variant test and SKAT WGS analyses using ASAFE.

Chr:position (hg38)	SNV/indel (Gene)	Test	Estimated ancestry-specific allele frequencies		
			African	European	Native American
19:3,799,817	rs539182790 (*MATK*)	Single variant test	3.1 × 10^−10^	7.9 × 10^−4^	2.2 × 10^−2^
16:79,599,332	rs1230233783 (*MAF)*	SKAT	1.4 × 10^−8^	4.2 × 10^−8^	2.0 × 10^−7^
11:102,593,550	rs149589493 (*MMP20*)	SKAT	7.4 × 10^−3^	2.4 × 10^−8^	8.9 × 10^−11^
6:44,376,382	rs190658489 (*SPATS1)*	SKAT	6.6 × 10^−4^	3.2 × 10^−8^	2.3 × 10^−7^
17:82,054,634	rs78902137 (*GPS1)*	SKAT	2.6 × 10^−2^	2.0 × 10^−8^	7.2 × 10^−16^

SNV, single nucleotide variant. *MMP20, SPATS1, GSP1* genes had suggestive association in gene-based SKAT analyses. rs1230233783 alternative allele is more common in East Asians (see text).

### 3.4 Replication

Chromosome 1 and 2 variants had higher allele frequencies in East Asians in the 1000 Genomes Project, so we attempted replication in two East Asian studies, the THRV study, a cohort of participants from Taiwan (*n* = 1,132) and the BBJ, a hospital-based study of 1,524 Japanese (Table 3). Although the p-values were not significant, there was consistent direction of effects between our data and the BBJ results for rs180996919 and rs116951054. We additionally attempted replication of the gene-based *MAF* findings in BBJ, which included 34 SNVs but the most influential missense SNV rs1230233783 was not avaialble. The gene-based associations were not significant for the burden test (*P* = 0.99) or SKAT test (*P* = 0.66).

**Table 3:** Replication findings for single variant test.

SNV, coded allele (gene)/ Replication study	Number	Minor Allele Count	Beta (SE)	*p*	Direction of effect consistency
rs180996919, C (*PRKAA2*)					
THRV	1,113	4	1.317 (2.853)	0.64	No
BBJ	1,524	6	−0.114 (0.305)	0.71	Yes
rs116951054, A (*METTL8*)					
THRV	1,113	7	−0.876 (2.039)	0.67	No
BBJ	1,524	4	0.274 (0.365)	0.45	Yes
rs539182790, G (*MATK*)					
HCHS/SOL Hispanics*	6,767	201	−0.963 (1.059)	0.36	Yes
Full Pima Indians	1,438	60	−0.013 (0.053)	0.81	Yes
Non-Full Pima Indians	757	17	0.080 (0.077)	0.30	No
FIND American Indians	836	56	0.026 (0.076)	0.73	No

BBJ, BioBank Japan; FIND, Family Investigation of Nephropathy and Diabetes; HCHS/SOL, Hispanic Community Health Study/Study of Latinos; NA, not available.

⁎ TOPMed freeze 5b imputed data.

We also attempted to replicate the Amerindian indel variant on chromosome 19 (rs539182790) in HCHS/SOL Hispanics/Latinos for individuals selected for high Amerindian ancestry (Mexican, Central American, and South American) (*n* = 6,578, MAF=0.01, *P* = 0.30), and among American Indians using several samples: participants of a community-based study, who are full heritage Pima Indians (*n* = 1,438, MAF=0.02, *P* = 0.86), non-full Pima Indians (*n* = 757, MAF=0.01, *P* = 0.30) and American Indian participants of the FIND study (about 1/3 Pima Indians and 2/3 other tribes, *n* = 836, MAF=0.03, *P* = 0.74). Although the p-values were not significant, the direction of effects was consistent with TOPMed for full heritage Pima Indians and HCHS/SOL Hispanic participants (Table 3).

### 3.5 Trans-ethnic eGFR loci reported in the genome catalog

Among 91 trans-ethnic identified variants present in our study, 53 were nominally associated with eGFR in our WGS data (including those reported on chromosomes 15 and 17). The most significant associations of known eGFR loci were for *GCKR* (*P* = 2.7 × 10^−8^), *UNCX* (*P* = 3.5 × 10^−8^), *VEGFA* (*P* = 1.2 × 10^−7^), *PRKAG2* (*P* = 2.8 × 10^−7^), *SHROOM3* (*P* = 2.6 × 10^−7^), *NFATC1* (*P* = 8.4 × 10^−7^), *MPPED2* (*P* = 8.9 × 10^−7^), *UMOD* (*P* = 1.3 × 10^−6^), *CPS1* (*P* = 4.4 × 10^−6^), *LRP2* (*P* = 3.0 × 10^−5^), *RGS14-SLC34A1* (*P* = 8.6 × 10^−5^) and *WDR37* (*P* = 8.7 × 10^−5^) (**Supp Table 3**). There was no evidence of secondary signals at these loci for further fine-mapping. We note that there is some overlap of our samples with published studies.

At the *UMOD* locus, the most significant associated SNV was rs77924615, an intronic variant of *PDILT* that has been identified in prior trans-ethnic GWAS meta-analyses of eGFR [7] (**Supp Fig 7a**). Additional SNVs at the promoter of *UMOD* have been identified in GWAS meta-analyses of eGFR in European ancestry (e.g. rs12917707). To investigate why SNVs did not achieve genome-wide significance at this widely replicated locus, we compared association estimates and p-values among European and non-European ancestry samples, noting that rs12917707 was not in linkage disequilibrium with rs77924615 in our data (**Supp Fig 7b**). These SNVs showed larger variance in estimates in non-European compared to European ancestry samples and had lower MAF in non-European compared to European ancestry data (**Supp Table 4**).

## 4 Discussion

This is the largest genetic study addressing a role of low frequency and rare variants on eGFR. Our study used deep coverage WGS (~38x) from five ancestral groups for a comprehensive assessment of SNVs across diverse populations. It also employed approaches suitable for analyses of rare variants among populations with ancestral admixture. By combining multi-ethnic groups, we optimised the power to detect low frequency alleles shared among ethnic groups with admixture, who may carry ancestry-specific rare variants. We accounted for recent and ancestral relatedness in these analyses, and genetic effects that are heterogeneous across ancestral groups, which are usually not addressed in GWAS. Lastly, we allowed for heterogeneity in eGFR distribution observed among ethnic groups, as African Americans showed larger eGFR trait variance than other groups. Using this strategy, we identified ancestry-specific low frequency variants influencing eGFR. We also confirmed associations for common variants at known loci, although this was not a main goal of this study. Importantly, our study uncovered several challenges for the study of rare ancestry-specific variants including finding suitable replication samples for validation of associations.

The main findings are related to 3 rare variants identified in single variant analyses, which showed a large effect on decreasing eGFR (chromosome 1) or increasing eGFR (chromosomes 2 and 19) and estimates ranging from 10 to 14 ml/min/1.73 m^2^. However, the PVE was small for each variant and their joined effects. Two identified SNVs more commonly observed in East Asians were located at *PRKAA2* (chromosome 1, rs180996919) and *METTL8* (chromosome 2, rs116951054, intronic). The *PRKAA2* SNV in intron 2 of the canonical mapped transcript (RefSeq NM_006252) is <500 bp 5′ to exon 3 of the gene. *PRKAA2* codes for the alpha2 isoform of the AMP-activated protein kinase (AMPK) subunit and knockdown of AMPKα2 has been shown to enhance the epithelial-mesenchymal transition, secretion of inflammatory factors, and concomitant fibrosis in proximal tubule cells in a mouse unilateral ureteral obstruction model, through up-regulation of beta-catenin and Smad3 [34]. Based on this empirical evidence, we hypothesize that the rare allele is leading to down-regulation of the total expression of the gene, or a differential regulation of a splice form involving the proximal exon. This SNV overlaps DHS sites in muscle, and it may affect creatinine production instead of kidney function. Little is known about *METTL8* but very recent work suggests that this gene is involved in mRNA editing through m^3^C epitranscriptomic processes, a potentially new mechanism of renal gene regulation [35]. Indeed, our functional annotation of the SNV supported a regulatory function in kidney (H3K4me1 broadPeak, an enhancer-associated mark in the Roadmap Epigenomics Consortium data). We were unable to replicate these associations given the paucity of ancestry-specific WGS samples for East Asians and the low frequency of these variants, with the variants not present in publicly available GWAS studies.

The chromosome 19 indel rs539182790 is a rare intronic SNV of the *MATK* gene. The MATK gene encodes for the megakaryocyte‐associated tyrosine kinase, which plays a role in the signal transduction of hematopoietic cells. Our SNV was identified as an Amerindian variant based on ancestry-specific allele frequency analyses. This SNV showed consistent direction of effect in replication samples of additional Hispanic individuals, and in one of two samples of southwest American Indians (Full Pima Indians) , although the replication p-values were not significant. Given the less known genetic architecture of American Indians and little relevant reference data for this population, we cannot rule out that the lack of replication of the Amerindian SNV is due to differences in ancestral backgrounds of Hispanic/Latino participants and the southwest American Indians who were used as replication.

Our findings also underscore the challenges to study low frequency variants in multi-ethnic studies and admixed populations when identified variants are specific to an ancestral group. Our gene-based analyses identified associations with eGFR at the *MAF* gene. *MAF* encodes a leucine zipper transcription factor, which has a role in embryological development of kidney cells [36]. The gene is highly expressed in adult kidney, with transcripts mapping to proximal tubule cells in healthy human kidney cells [37]. A common variant near *MAF* was associated with uric acid levels in East Asians [33] and it was associated with accelerated eGFR decline among East Asian diabetic subjects [38]. The most influential SNV at *MAF* gene-based analysis, rs1230233783, is a missense variant with predicted deleterious effects based on multiple annotation algorithms including a Combined Annotation Dependent Depletion (CADD) score of 13.4. This SNV has both regulatory and coding-effect annotations (H3K27ac peak in the ENCODE data), and further functional studies are needed to assess its relevance in the context of human disease genomics. However, additional studies are needed to confirm our findings as we were not able to identify a suitable large sample for replication of the SNV.

We also identified differences in allele frequency and a larger variance in effect estimates for two well-replicated *UMOD* common variants when comparing non-European to European ancestry samples, which may explain the lack of genome-wide association significance at this locus in our multi-ethnic sample. Allele heterogeneity across ancestries may also contribute to these findings.

To our knowledge, this is the first multi-ethnic WGS of eGFR. A prior study from Iceland performed WGS in 2,230 individuals and imputed sequenced variants into 81,656 chip genotyped individuals [19]. This study identified low frequency missense and LoF variants associated with serum creatinine at the *SLC6A19, SLC25A45, SLC47A1, RNF186* and *RNF128* genes. The study was restricted to individuals of European ancestry. A gene-based analyses of eGFR of variants in the exome array identified associations at the *SOS2* gene in individuals mostly of European ancestry [39]*.* These genes were not significant in our gene-based analyses.

Rare and low frequency variants are more likely to be population-specific and their genetic contribution to eGFR variation is mostly unknown. Our study provides important information for future WGS studies of rare SNVs for kidney traits, with implications for study design of SNV discovery and replication, particularly when studying diverse populations. An important contribution of this study is the application of a recently developed method to identify suitable replication populations for ancestry-specific variants identified in WGS, for SNVs that may not be available in public repositories and/or have unknown frequency in populations. Using ASAFE, we used local ancestry to estimate the allele frequency of our significant variants across populations and determined that the chromosome 19 variant is Amerindian, while the most common variant at the *MAF* gene is more common in East Asians. We expect that this approach will help to guide replication efforts for WGS studies of complex traits in multi-ethnic studies or admixed populations. Local ancestry in admixture mapping approaches could provide additional discovery when large WGS samples are available in multi-ethnic populations.

In summary, we performed a comprehensive genome-wide discovery study of eGFR in multi-ethnic studies using WGS of over 23,000 individuals that included association and admixture mapping approaches. We identified ancestry-specific low frequency variants associated with eGFR in both single variant test and gene-based analyses and used estimated local ancestry to guide replication of findings. Our study exemplifies the challenges of studying diverse populations including finding suitable replication samples for ancestry-specific low frequency variants identified in multi-ethnic studies and admixed populations. In addition, resources for functional characterization of these identified WGS rare variants are currently not available.

## Data sharing statement

All WGS TOPMed data and phenotype data are available at the dbGap access #: phs000956 (Amish), phs000964 (JHS), phs000974 (FHS), phs001211 (ARIC), phs001217 (GenSalt), phs001218 (GeneSTAR), phs001237 (WHI), phs001293 (HyperGEN), phs001345 (GENOA), phs001368 (CHS), phs001387 (THRV), phs001395 (HCHS/SOL), phs001416 (MESA), phs001612 (CARDIA).

## Funding sources

Additional funding sources are shown in Supplemental Data

## Web resources

Analysis Commons, http://analysiscommons.com

GENESIS, https://github.com/UW-GAC/GENESIS

Omics Analysis, Search and Information System (OASIS), https://omicsoasis.github.io

TOPMed Guidelines for Reporting Race/Ethnicity and Ancestry, https://www.nhlbiwgs.org/guidelines-use-and-reporting-race-ethnicity-and-ancestry-topmed

TOPMed Pipeline, https://www.nhlbiwgs.org/topmed-whole-genome-sequencing-project-freeze-5b-phases-1-and-2

TOPMed website, htpps://www.nhlbiwgs.org

TOPMed full authorship list, htpps://www.nhlbiwgs.org/topme-banner-authorship

WGSAPARSR 6.2.4, http://doi.org/10.5281/zenodo.3352386

## Authors contribution

Study design: NF, DL, BML, KEG, JCM, SRB. Data Collection: XG, BHD, ACH, SH, SK, JPL, SKM, APR, ARS, RBW, KY, SSakaue, KM, YK, YM, LRY, BMP, DKA, EB, JC, Y-DIC, AC, JH, SLRK, CK, RAM, BDM, DAN, STT, VSR, JIR, DLevy, SSR, LF. Data analyses: BML, KEG, JCM, LJB, YO, NF. Computational/statistical resources: JAB, CEB, TAT, JAP, DJ, YL, AVS, GA, LAC, DL Drafting of manuscript: BML, KEG, JCM, SRB, NF. Data interpretation: BLM, KEG, JAB, LMR, JCM, CEB, JAP, LJB, SSR. All authors reviewed and approved the manuscript.

Members of the NHLBI Trans-Omics for Precision Medicine (TOPMed) Consortium and the TOPMed Kidney Working Group

Bridget M Lin, Kelsey E Grinde, Jennifer A Brody, Charles E Breeze, Laura M Raffield, Josyf C Mychaleckyj, Timothy A Thornton, James A Perry, Lisa de las Fuentes, Xiuqing Guo, Benjamin D Heavner, Yi-Jen Hung, Huijun Qian, Chao A Hsiung, Shih-Jen Hwang, Margaret R Irvin, Deepti Jain,  Tanika N Kelly, Leslie Lange, James P Lash, Yun Li, Xiaoming Liu, Xuenan Mi, Solomon K Musani, George J Papanicolaou, Afshin Parsa, Alex P Reiner, Shabnam Salimi, Wayne H—H Sheu, Alan R Shuldiner, Kent D Taylor, Albert V Smith, Jennifer A Smith, Adrienne Tin, Dhananjay Vaidya, Robert B Wallace, Lisa R Yanek, Betsi A Young, Wei Zhao, Gonzalo Abecasis, Bruce M Psaty, Donna K Arnett, Eric Boerwinkle, Jianwen Cai, Ida Yii-Der Chen, Adolfo Correa, L Adrienne Cupples, Jiang He, Sharon LR Kardia, Charles Kooperberg, Rasika A Mathias, Braxton D Mitchell, Deborah A Nickerson, Steve T Turner, Vasan S Ramachandran, Jerome I Rotter, Daniel Levy, Holly J Kramer, Anna Köttgen, Stephen S Rich, Dan-Yu Lin, Sharon R Browning, Nora Franceschini.

## Declaration of Competing Interest

GRA is employed by Regeneron Pharmaceuticals and he owns stock and stock options for Regeneron Pharmaceuticals. BMP serves on the Steering Committee of the Yale Open Data Access Project funded by Johnson & Johnson. BMP reports serving on the Steering Committee of the Yale Open Data Access Project funded by Johnson & Johnson. Y-DIC, LRY, JCM, BDM, JIR, KDT, JPL, EB, JAS, GRA report grants from NIH during the conduct of the study. Remaining authors have nothing to disclose.

## Appendix Supplementary materials

Image, application 1

Image, application 2
