# Supplementary material for: Whole genome sequence analyses of eGFR in 23,732 people representing multiple ancestries in the NHLBI trans-omics for precision medicine (TOPMed) consortium
Source: eBioMedicine. 2021 Jan 6;63:103157. doi: 10.1016/j.ebiom.2020.103157 (PMC7804602; doi:10.1016/j.ebiom.2020.103157)
Supplement: Supplementary file 2 [file mmc2.docx]

**Whole genome sequence analyses of eGFR in 23,732 people representing multiple ancestries in the NHLBI Trans-Omics for Precision Medicine (TOPMed) Consortium**

Supp Fig 1-7

Supp Tables 1-2, and 4, Supp Table 3 in an excel file.

Variance explained by newly associated SNVs

Acknowledgements

**Supp Fig 1.** Quantile-quantile (QQ) plots for (a) single variant test (b) SKAT test (c) Burden test

**a. b.**


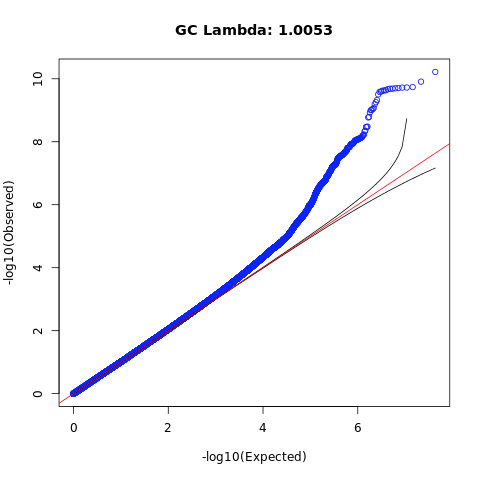

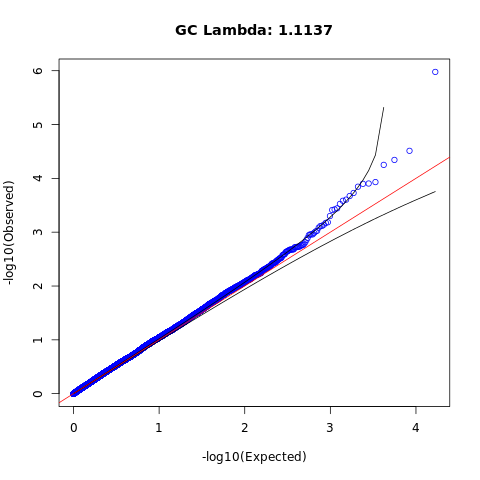


**c.**


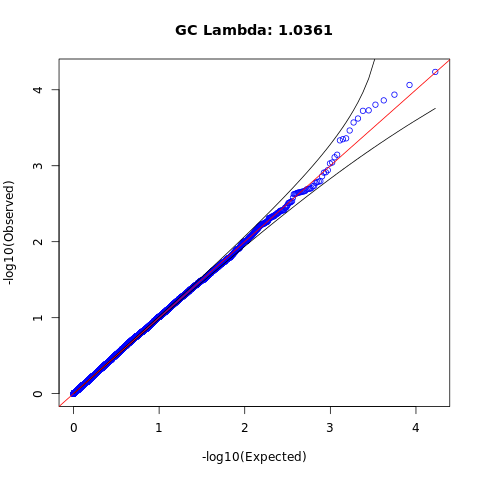


**Supp Fig 2.** Regional plots for conditional analyses on the most significant SNV at the chromosome 15 (*GATM*) and chromosome 17 (*CDK12*) loci.


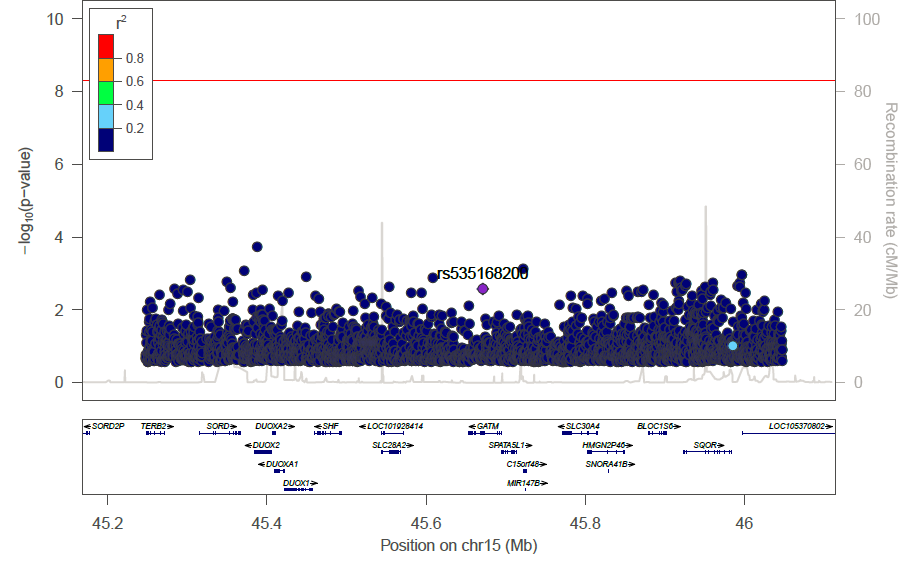


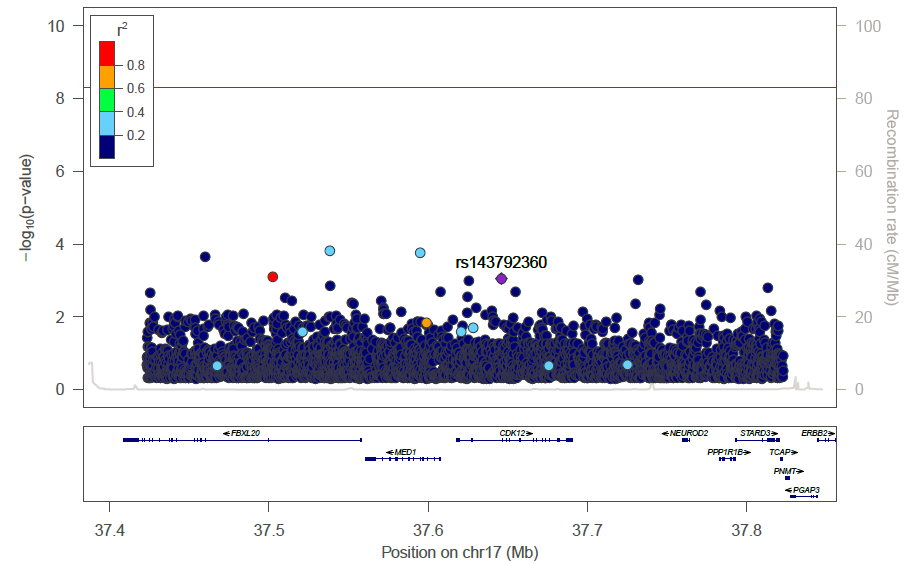


**Supp Fig 3.** Barplots of estimated admixture proportions in admixed TOPMed samples. (a) Estimated proportions of African (orange), European (blue), and Native American (grey) ancestry for self-identified African American TOPMed samples included in our admixture mapping analysis. (b)  Estimated proportions of African (orange), European (blue), and Native American (grey) ancestry for self-identified Hispanic American TOPMed samples included in our admixture mapping analysis.

a

b**
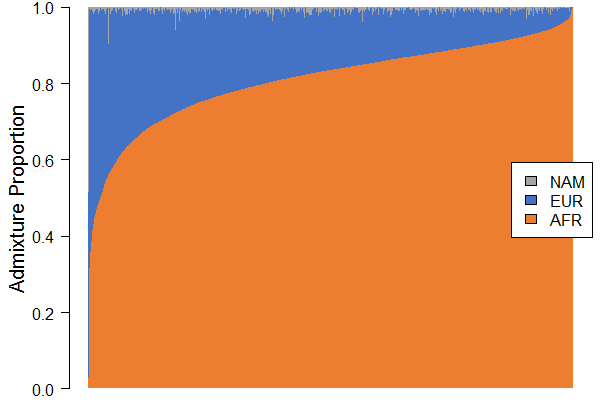

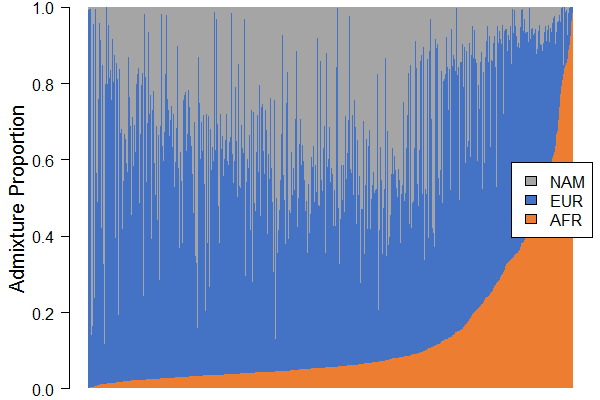
**

| Self-reported/inferred global ancestry | African | European | Native American |
| --- | --- | --- | --- |
| African American | 0.807 (0.001 - 0.997) | 0.182 (0.003 - 0.993) | 0.011 (0.000 - 0.485) |
| Hispanic/Latino | 0.136 (0.000 - 0.984) | 0.551 (0.004 - 0.994) | 0.313 (0.000 - 0.996) |

**Supp Fig 4.** Manhattan plots for eGFR admixture mapping study using 9,479 African American and Hispanic/Latino TOPMed samples. The three panels present results investigating the association between eGFR and African (top panel, $\lambda=1.082$), European (middle panel, $\lambda=0.992$), or Native American (bottom panel, $\lambda=0.954$) local ancestry. The dashed line represents the genome-wide p-value threshold of 5.4×10^−6^.


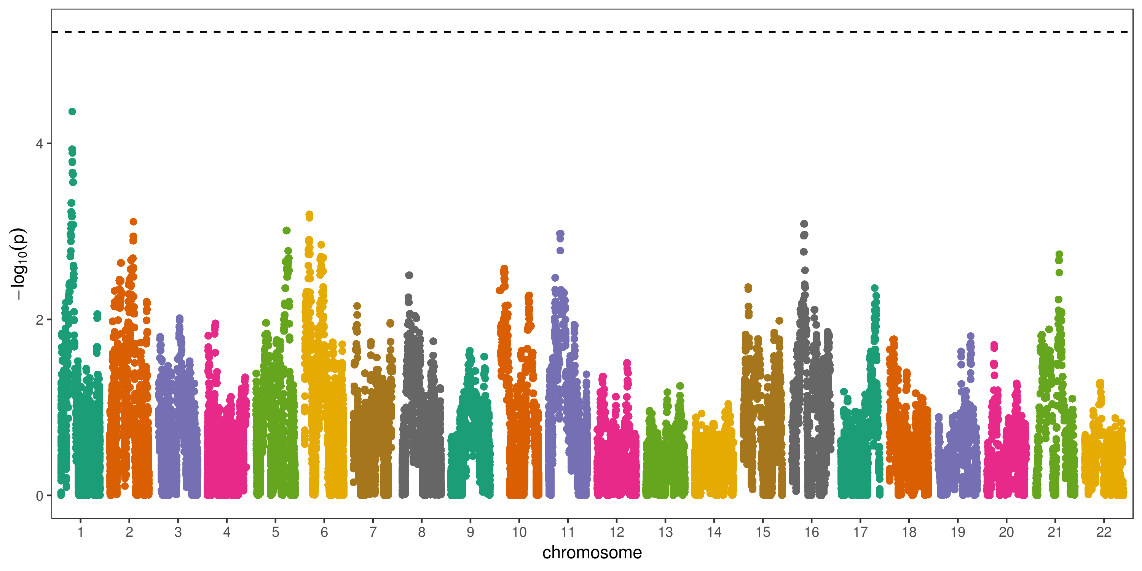

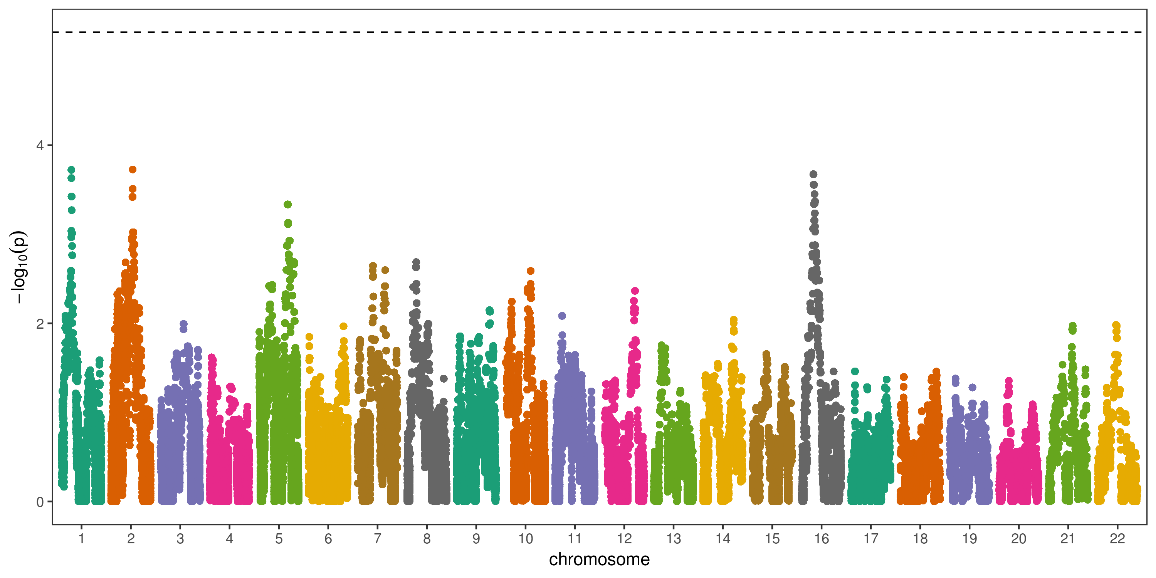

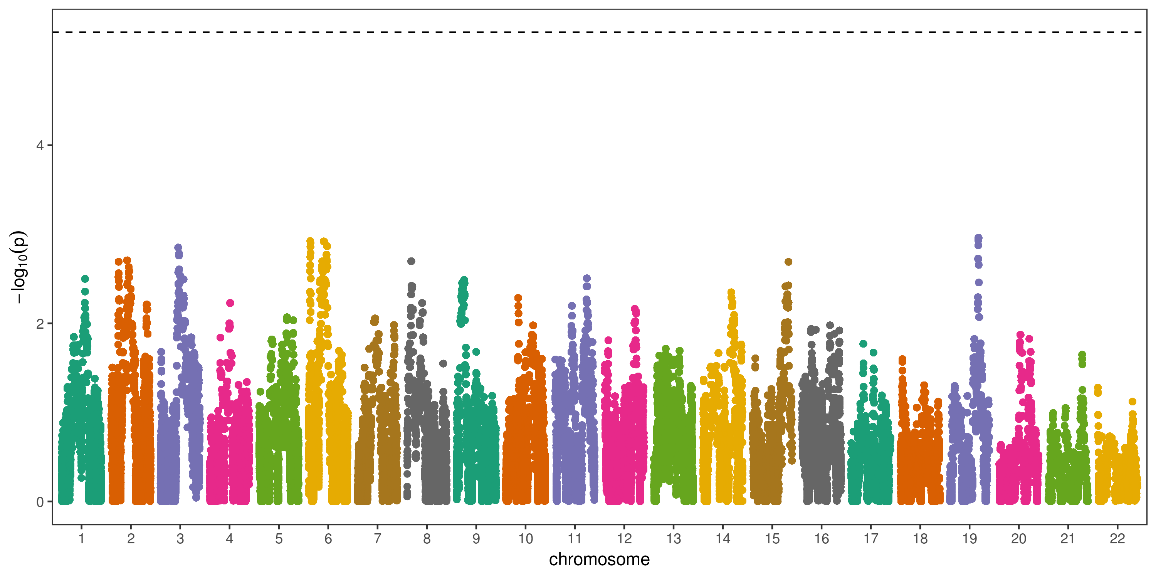


**Supp Fig 5.** Manhattan plots for eGFR admixture mapping study using 8,303 African American TOPMed samples. In this analysis, we only tested African ancestry ($\lambda=1.022)$. The dashed line represents the genome-wide p-value threshold of 1.6×10^-5^.


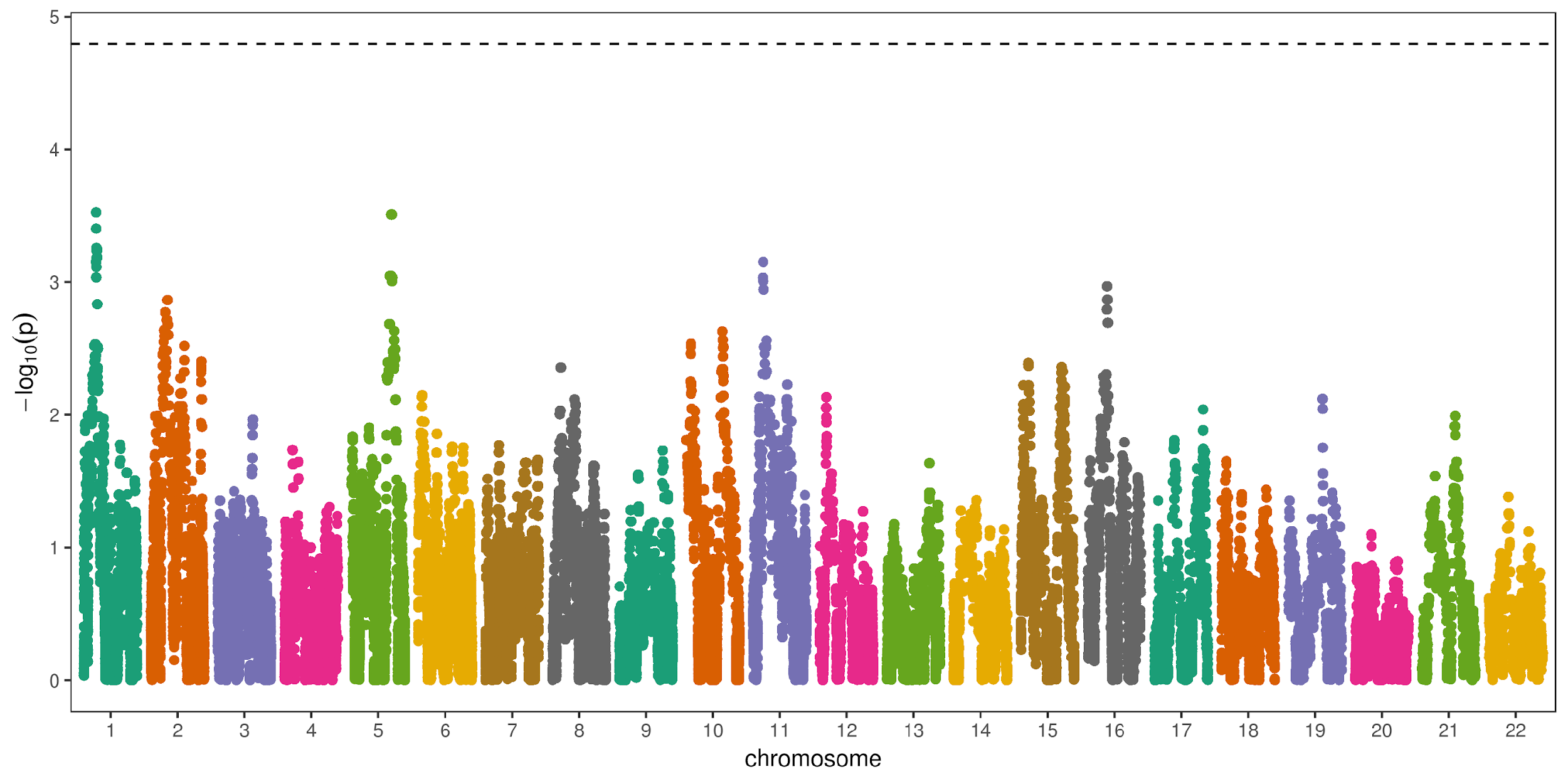


**Supp Fig 6.** Manhattan plots for eGFR admixture mapping study using 1,176 Hispanic/Latino TOPMed samples. The three panels present results investigating the association between eGFR and African (top panel, $\lambda=1.143$), European (middle panel, $\lambda=0.951$), or Native American (bottom panel, $\lambda=1.035$) local ancestry. The dashed line represents the genome-wide p-value threshold of 3.5×10^-6^.


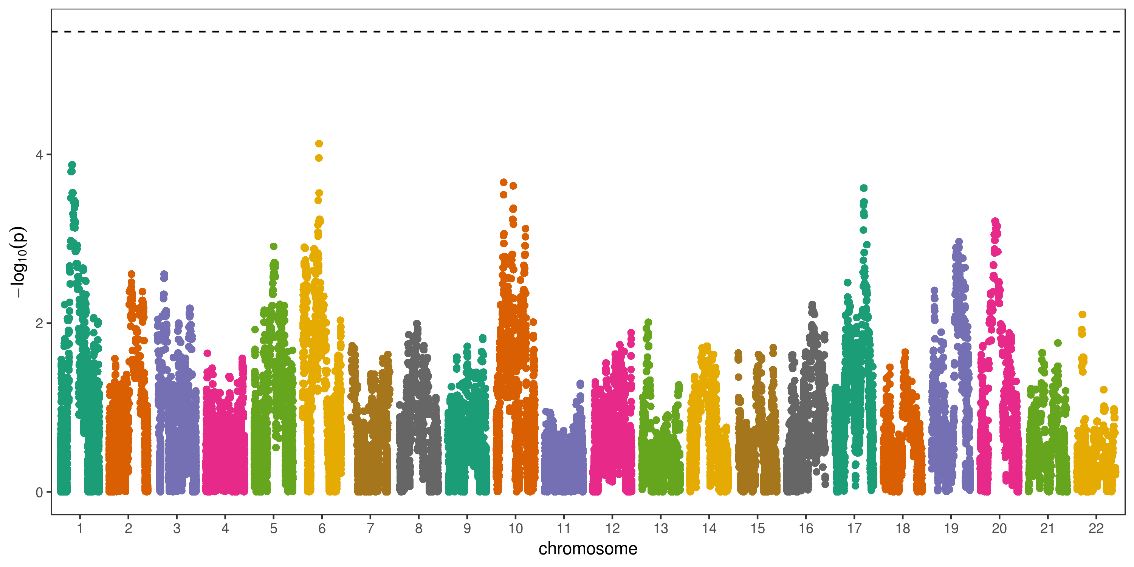


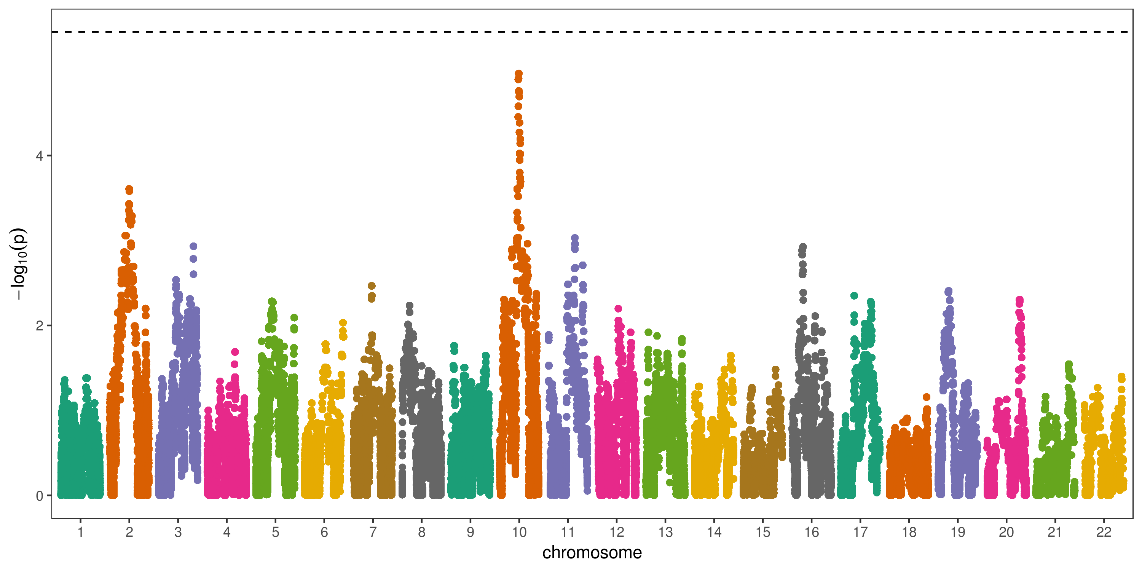


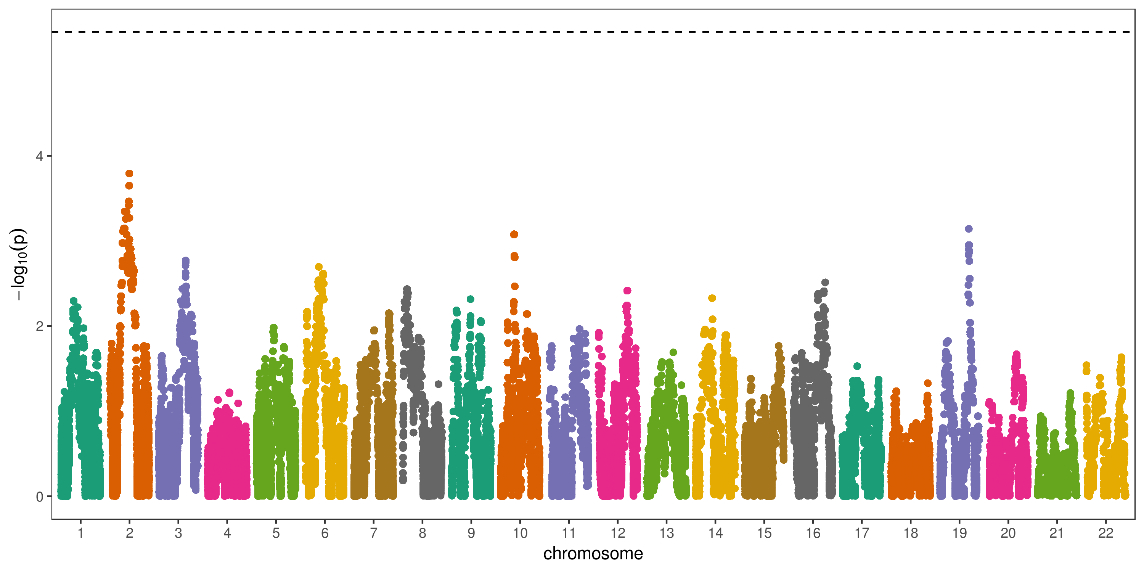


**Supp Fig 7.** Regional plot for association at the *UMOD* locus showing (a) the most significantly associated variant in our data and (b) the rs12917707 variant at *UMOD*. Note these variants are not in linkage disequilibrium in our data.

a


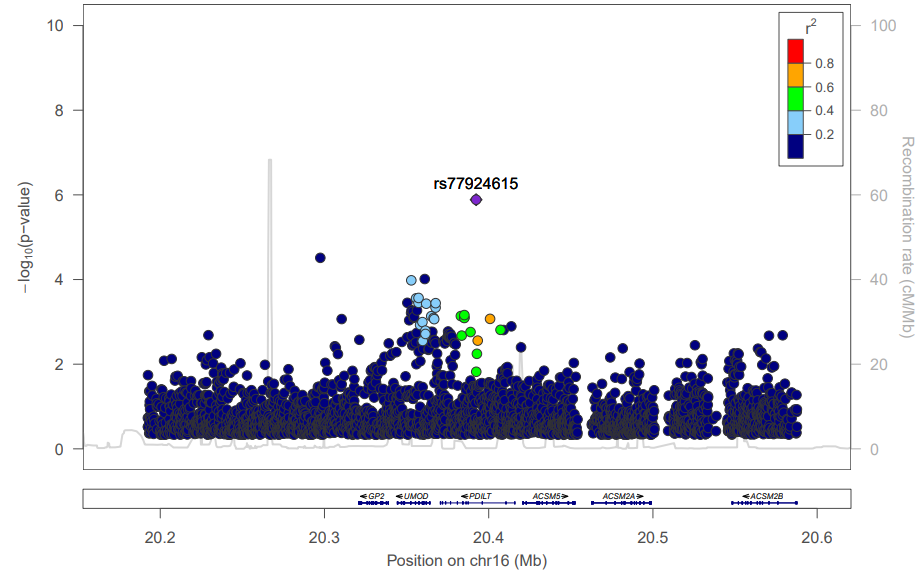


b


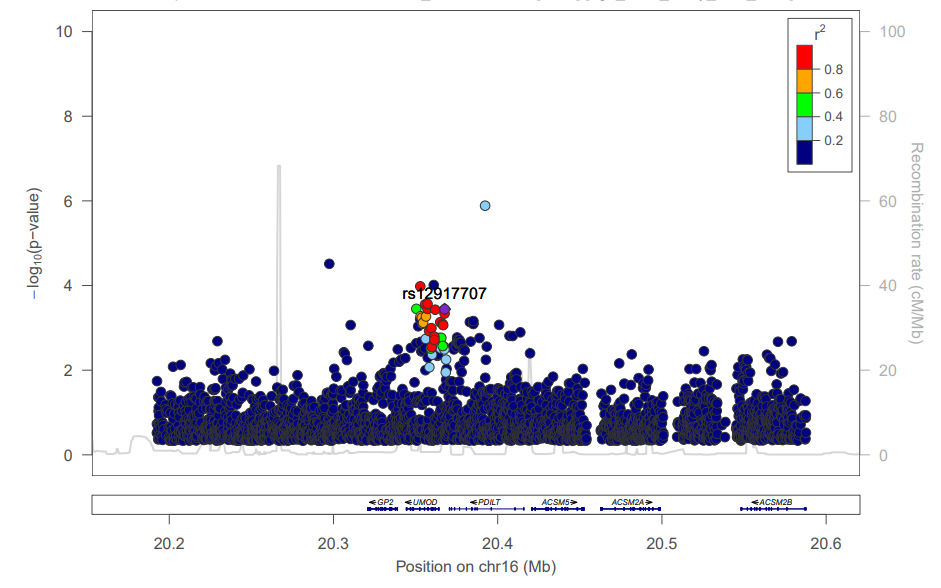


**Supp Table 1.** Descriptive analyses of TOPMed participants by racial/ethnic groups. Note that we tested association in combined samples, while using an ancestry-adjusted kinship and allowing for heterogeneous variance within racial/ethnic groups

| Characteristics | African American | Hispanic/Latino | East Asian | European American | Native American |
| --- | --- | --- | --- | --- | --- |
| Total number | 8459 | 1182 | 2208 | 11837 | 46 |
| Mean age (SD) | 55.0 (12.7) | 61.0 (9.4) | 44.2 (13.7) | 57.8 (12.1) | 63.6 (7.4) |
| Male sex % | 32.20 | 38.66 | 52.45 | 37.15 | 0 |
| Mean eGFR (SD) | 92.2 (22.2) | 85.9 (18.5) | 95.0 (18.6) | 91.7 (16.9) | 85.2 (15.3) |
| Diabetes % | 23.1 | 8.7 | 13.4 | 9.9 | 17.4 |
| Hypertension % | 54.0 | 33.8 | 10.1 | 27.1 | 0.5 |
| eGFR<60 ml/min/1.73 m^2^ % | 7.32 | 4.74 | 2.36 | 4.06 | 8.70 |

eGFR, estimated glomerular filtration rate; SD, standard deviation

**Supp Table 2.** Main findings for SKAT gene-based analyses for eGFR and corresponding p-values from gene-based burden test

| Ensembl/gene name | Gene abbreviation (name) | Chr:position (hg38) | Number of SNV within genes | n.sample.alt | SKAT *p* | Burden *p* |
| --- | --- | --- | --- | --- | --- | --- |
| ENSG00000178573 | *MAF* (MAF bZIP transcription factor) | 16:79599017 | 61 | 568 | 1.1 x 10^-6^ | 3.1 x 10^-3^ |
| ENSG00000169727 | *GPS1 (*G protein pathway suppressor 1) | 17:82056475 | 55 | 812 | 3.1 x 10^-5^ | 2.4 x 10^-2^ |
| ENSG00000137674 | *MMP20 (*matrix metallopeptidase 20) | 11:102593438 | 22 | 157 | 4.5 x 10^-5^ | 3.4 x 10^-2^ |
| ENSG00000249481 | *SPATS1 (*spermatogenesis associated serine rich 1) | 6:44360571 | 8 | 16 | 5.6 x 10^-5^ | 3.1 x 10^-2^ |

Only *MAF* association is significant at gene-based level.

SNV, single nucleotide variant; *p*, p-value; n.sample.alt, the number of samples with an observed alternate allele at any variant in the aggregate set

**Supp Table 3.** See excel file

**Supp Table 4.** Associations of two variants at the *UMOD* locus among European and non-European samples, and in combined trans-ethnic analyses. Note differences in allele frequencies for SNVs comparing European to non-European samples and differences in the variance of effect estimates for associations with eGFR.

European:

| SNV-coded allele | N | freq | Effect (SE) | P | ref | alt | snpID |
| --- | --- | --- | --- | --- | --- | --- | --- |
| rs12917707-T | 11837 | 0.19 | 0.4142 (0.1201) | 0.0010 | G | T | 16:20356368:G:T |
| rs77924615-A | 11837 | 0.20 | 0.5212 (0.1172) | 0.0017 | G | A | 16:20381010:G:A |

No European:

| SNV-coded allele | N | freq | Effect (SE) | P | ref | alt | snpID |
| --- | --- | --- | --- | --- | --- | --- | --- |
| rs12917707-T | 11895 | 0.05 | 0.4337 (0.3283) | 0.00015 | G | T | 16:20356368:G:T |
| rs77924615-A | 11895 | 0.10 | 0.4783 (0.2388) | 0.00034 | G | A | 16:20381010:G:A |

Combined European and non-European trans-ethnic analyses:

| SNV-coded allele | N | freq | Effect (SE) | P | ref | alt | snpID |
| --- | --- | --- | --- | --- | --- | --- | --- |
| rs12917707-T | 23732 | 0.12 | 0.4103 (0.1150) | 3.61E-04 | G | T | 16:20356368:G:T |
| rs77924615-A | 23732 | 0.15 | 0.5179 (0.1070) | 1.30E-06 | G | A | 16:20381010:G:A |

**Variance explained by newly associated SNVs**

For single SNP Tests in a meta-population, where the meta-covariates are modeled as $X\beta$and the variance explained by the SNP under test is *V_i_*:

|  | $Var(Y) = Var(X\beta{) + V}_{i}+\sigma_{i}^{2}$ | (1) |
| --- | --- | --- |

The phenotypic variance not explained by the covariates for each SNP is:

|  | $Var(Y) - Var(X\beta{)= V}_{1}+\sigma_{1}^{2}=V_{2}+\sigma_{2}^{2}=....{= V}_{K}+\sigma_{K}^{2}$ | (2) |
| --- | --- | --- |

This assumes the usual conditions on applicability of the linear models and that variation explained by each SNP is independent of the covariates. In addition to assuming the independence of each SNP and the covariates, if the *K* SNPs of interest are also assumed mutually independent, i.e. no SNP-SNP LD or residual covariance due to unadjusted population stratification:

|  | $\sigma_{T}^{2}+ \sum_{i=i}^{K} V_{i}{= V}_{1}+\sigma_{1}^{2}=V_{2}+\sigma_{2}^{2}=....{= V}_{K}+\sigma_{K}^{2}$ | (3) |
| --- | --- | --- |

where $\sigma_{T}^{2}$ is the residual unexplained variance if all K SNPs were jointly modelled. Again assuming that the meta-population contains no residual population structure so that a single homogeneous allele frequency reasonably approximates the allele distribution and further, that the SNP is in Hardy-Weinberg equilibrium in the meta-population, $\hat{V}_{i}$ for a single SNP can be estimated as:

|  | $\hat{V}_{i} \sim{2\hat{\beta}}_{i}^{2}\hat{f}(1-\hat{f})$ | (4) |
| --- | --- | --- |

where $\hat{f_{i}}$ is the allele frequency. From the standard least squares estimate of var($\hat{\beta}_{i})$:

|  | ${var(\hat{\beta}_{i}) =[se(\hat{\beta}_{i})]}^{2} \sim\frac{{\hat{\beta}_{i}^{2} \hat{\sigma}}_{i}^{2}}{N \hat{V}_{i}}$ | (5) |
| --- | --- | --- |

where *N* is the sample size. Rearranging:

|  | $\hat{\sigma}_{i}^{2}= \frac{{N \hat{V}_{i}[se(\hat{\beta}_{i})]}^{2}}{\hat{\beta}_{i}^{2}} = \frac{N \hat{V}_{i}}{\hat{Z}_{i}^{2}}$ | (6) |
| --- | --- | --- |

where *Z_i_* is the test statistic and $\hat{Z}_{i}^{2} \sim\chi_{1}^{2}$ under the null as N becomes large. Taking the mean of the K estimates from (3) :

|  | $\hat{\sigma}_{T}^{2}=\frac{1}{K}\sum_{i=i}^{K} \hat{\sigma}_{i}^{2} + \frac{1-K}{K}\sum_{i=i}^{K} \hat{V}_{i}$ | (7) |
| --- | --- | --- |

$\hat{\sigma}_{T}^{2}$ can be estimated from (4) and (6) for the individual SNPs. Then the total PVE for all K SNPs jointly is:

|  | $PVE (K SNPs) = \frac{\sum_{i=i}^{K} \hat{V}_{i}}{\sum_{i=i}^{K} \hat{V}_{i}+ \hat{\sigma}_{T}^{2}}$ | (8) |
| --- | --- | --- |

| chr | pos | MAC | Score | Score.SE | Score.Stat | Score.pval | Est | Est.SE | PVE |
| --- | --- | --- | --- | --- | --- | --- | --- | --- | --- |
| 1 | 56690933 | 19 | -2.93433 | 0.44890 | -6.53667 | 6.29057 | -14.56137 | 2.22764 | 0.00180195638924101 |
| 19 | 3799817 | 26 | -3.60026 | 0.60955 | -5.90634 | 3.4978e-09 | -9.68954 | 1.64053 | 0.0014711921456981 |
| 2 | 171363037 | 41 | 3.77524 | 0.64406 | 5.86157 | 4.5852e-09 | 9.10087 | 1.55263 | 0.00144896957997609 |

We compute the total PVE for 1: 56690933, 19: 3799817, and 2: 171363037.

$$\hat{\sigma}_{T}^{2}=\frac{1}{K}\sum_{i=i}^{K} \hat{\sigma}_{i}^{2} + \frac{1-K}{K}\sum_{i=i}^{K} \hat{V}_{i}=\frac{1}{K}\sum_{i=i}^{K} \frac{N \hat{V}_{i}}{\hat{Z}_{i}^{2}} + \frac{1-K}{K}\sum_{i=i}^{K} \hat{V}_{i}$$

$$=\frac{1}{3}*\left( \frac{23732* 0.00180196}{({-6.53667)}^{2}}+\frac{23732* 0.00147119}{({-5.90634)}^{2}}+\frac{23732* 0.00144896}{(5.86157)^{2}} \right)$$

$$+ \frac{1-3}{3}*(0.00180196+0.00147119+0.00144896)$$

$$=0.997693$$

$$PVE \left( 3 SNPs \right)= \frac{\sum_{i=i}^{K} \hat{V}_{i}}{\sum_{i=i}^{K} \hat{V}_{i}+ \hat{\sigma}_{T}^{2}}= \frac{0.00180196+0.00147119+0.00144896}{0.00180196+0.00147119+0.00144896+0.997693}$$

$$=0.004710733$$

**Acknowledgments**

Whole genome sequencing centers by study are shown in **Acknowledgment Table**. Centralized read mapping and genotype calling, along with variant quality metrics and filtering were provided by the TOPMed Informatics Research Center (3R01HL-117626-02S1; contract HHSN268201800002I).  Phenotype harmonization, data management, sample-identity QC, and general study coordination were provided by the TOPMed Data Coordinating Center (3R01HL-120393-02S1; contract HHSN268201800001I). We gratefully acknowledge the studies and participants who provided biological samples and data for TOPMed.

*Study specific acknowledgements*

Old Order Amish: This work was supported by NIH grant R01 HL121007.

The Atherosclerosis Risk in Communities study has been funded in whole or in part with Federal funds from the National Heart, Lung, and Blood Institute, National Institutes of Health, Department of Health and Human Services (contract numbers HHSN268201700001I, HHSN268201700002I, HHSN268201700003I, HHSN268201700004I and HHSN268201700005I). The authors thank the staff and participants of the ARIC study for their important contributions.

The Framingham Heart Study (FHS) acknowledges the support of Contracts NO1-HC-25195, HHSN268201500001I and 75N92019D00031 from the National Heart, Lung and Blood Institute and grant supplement R01 HL092577-06S1 for this research. We also acknowledge the dedication of the FHS study participants without whom this research would not be possible.

GeneSTAR was supported by the National Institutes of Health/National Heart, Lung, and Blood Institute (U01 HL72518, HL087698, HL112064, HL58625) and by a grant from the National Institutes of Health/National Center for Research Resources (M01-RR000052) to the Johns Hopkins General Clinical Research Center.

Support for GENOA was provided by the National Heart, Lung and Blood Institute (HL054457, HL054464, HL054481, HL087660, and HL119443).

The Genetic Epidemiology Network of Salt-Sensitivity (GenSalt) was supported by research grants (U01HL072507, R01HL087263, and R01HL090682) from the National Heart, Lung and Blood Institute, National Institutes of Health, Bethesda, MD. Research reported in this publication was also supported by the National Institute of General Medical Sciences of the National Institutes of Health under Award Number P20GM109036.

The Hispanic Community Health Study/Study of Latinos was carried out as a collaborative study supported by contracts from the National Heart, Lung, and Blood Institute (NHLBI) to the University of North Carolina (N01-HC65233), University of Miami (N01-HC65234), Albert Einstein College of Medicine (N01-HC65235), Northwestern University (N01-HC65236), and San Diego State University (N01-HC65237). The following Institutes/Centers/Offices contribute to the HCHS/SOL through a transfer of funds to the NHLBI: National Center on Minority Health and Health Disparities, the National Institute of Deafness and Other Communications Disorders, the National Institute of Dental and Craniofacial Research, the National Institute of Diabetes and Digestive and Kidney Diseases, the National Institute of Neurological Disorders and Stroke, and the Office of Dietary Supplements.

The HyperGEN Study is part of the National Heart, Lung, and Blood Institute (NHLBI) Family Blood Pressure Program; collection of the data represented here was supported by grants U01 HL054472 (MN Lab), U01 HL054473 (DCC), U01 HL054495 (AL FC), and U01 HL054509 (NC FC). The HyperGEN: Genetics of Left Ventricular Hypertrophy Study was supported by NHLBI grant R01 HL055673 with whole-genome sequencing made possible by supplement -18S1.

The Jackson Heart Study (JHS) is supported and conducted in collaboration with Jackson State University (HHSN268201300049C and HHSN268201300050C), Tougaloo College (HHSN268201300048C), and the University of Mississippi Medical Center (HHSN268201300046C and HHSN268201300047C) contracts from the National Heart, Lung, and Blood Institute (NHLBI) and the National Institute for Minority Health and Health Disparities (NIMHD). The authors also wish to thank the staffs and participants of the JHS.

The acknowledgements  for MESA will also need to be updated to read:

MESA and the MESA SHARe project are conducted and supported by the National Heart, Lung, and Blood Institute (NHLBI) in collaboration with MESA investigators. Support for MESA is provided by contracts 75N92020D00001, HHSN268201500003I, N01-HC-95159, 75N92020D00005, N01-HC-95160, 75N92020D00002, N01-HC-95161, 75N92020D00003, N01-HC-95162, 75N92020D00006, N01-HC-95163, 75N92020D00004, N01-HC-95164, 75N92020D00007, N01-HC-95165, N01-HC-95166, N01-HC-95167, N01-HC-95168, N01-HC-95169, UL1-TR-000040, UL1-TR-001079, UL1-TR-001420. The provision of genotyping data was supported in part by the National Center for Advancing Translational Sciences, CTSI grant UL1TR001881, and the National Institute of Diabetes and Digestive and Kidney Disease Diabetes Research Center (DRC) grant DK063491 to the Southern California Diabetes Endocrinology Research Center.

The Rare Variants for Hypertension in Taiwan Chinese (THRV) is supported by the National Heart, Lung, and Blood Institute (NHLBI) grant (R01HL111249) and its participation in TOPMed is supported by an NHLBI supplement (R01HL111249-04S1). THRV is a collaborative study between Washington University in St. Louis, LA BioMed at Harbor UCLA, University of Texas in Houston, Taichung Veterans General Hospital, Taipei Veterans General Hospital, Tri-Service General Hospital, National Health Research Institutes, National Taiwan University, and Baylor University. THRV is based (substantially) on the parent SAPPHIRe study, along with additional population-based and hospital-based cohorts. SAPPHIRe was supported by NHLBI grants (U01HL54527, U01HL54498) and Taiwan funds, and the other cohorts were supported by Taiwan funds.

The WHI program is funded by the National Heart, Lung, and Blood Institute, National Institutes of Health, U.S. Department of Health and Human Services through contracts HHSN268201600018C, HHSN268201600001C, HHSN268201600002C, HHSN268201600003C, and HHSN268201600004C

Studies of the southwest American Indians were supported by the Intramural Program of NIDDK.

**Acknowledgement Table.** TOPMed Study Sequencing Support

| **TOPMed Accession #** | **Parent Study Short Name** | **Parent Study Full Name** | **TOPMed Phase** | **TOPMed Project** | **Omics Center** | **Omics Support Grant/Contract Number** |
| --- | --- | --- | --- | --- | --- | --- |
| phs000956 | Amish | Genetics of Cardiometabolic Health in the Amish | CCDG co-funded | AFGen | BROAD | 3R01HL121007-01S1 |
| phs001211 | ARIC | Atherosclerosis Risk in Communities Study | 1 | AFGen | BROAD | 3R01HL092577-06S1 |
| phs001211 | ARIC | Atherosclerosis Risk in Communities Study VTE cohort | 2 | VTE | BAYLOR | 3U54HG003273-12S2, HHSN268201500015C |
| phs000974 | FHS | Framingham Heart Study | 1 | FHS | BROAD | 3U54HG003067-12S2 |
| phs001218 | GeneSTAR | Genetic Studies of Atherosclerosis Risk | 2 | AA_CAC | BROAD | HHSN268201500014C |
| phs001218 | GeneSTAR | Genetic Studies of Atherosclerosis Risk | 2 | GeneSTAR | MACROGEN | 3R01HL112064-04S1 |
| phs001218 | GeneSTAR | Genetic Studies of Atherosclerosis Risk | legacy | GeneSTAR | ILLUMINA | R01HL112064 |
| phs001345 | GENOA | Genetic Epidemiology Network of Arteriopathy | 2 | AA_CAC | BROAD | HHSN268201500014C |
| phs001345 | GENOA | Genetic Epidemiology Network of Arteriopathy | 2 | HyperGEN_GENOA | UW NWGC | 3R01HL055673-18S1 |
| phs001217 | GenSalt | Genetic Epidemiology Network of Salt Sensitivity | 2 | GenSalt | BAYLOR | HHSN268201500015C |
| phs001293 | HyperGEN | Hypertension Genetic Epidemiology Network | 2 | HyperGEN_GENOA | UW NWGC | 3R01HL055673-18S1 |
| phs000964 | JHS | Jackson Heart Study | 1 | JHS | UW NWGC | HHSN268201100037C |
| phs001416 | MESA | Multi-Ethnic Study of Atherosclerosis | 2 | MESA | BROAD | 3U54HG003067-13S1 |
| phs001387 | THRV | Taiwan Study of Hypertension using Rare Variants | 2 | THRV | BAYLOR | 3R01HL111249-04S1, HHSN26820150015C |
| phs001237 | WHI | Women's Health Initiative | 2 | WHI | BROAD | HHSN268201500014C |
